# Recent Advances in Plant Nanoscience

**DOI:** 10.1002/advs.202103414

**Published:** 2021-11-10

**Authors:** Qi Zhang, Yibin Ying, Jianfeng Ping

**Affiliations:** ^1^ Laboratory of Agricultural Information Intelligent Sensing College of Biosystems Engineering and Food Science Zhejiang University Hangzhou 310058 P. R. China

**Keywords:** nanocarriers, nanofertilizers, nanogenerators, nanopesticides, nanosensors, nanotoxicology, plant nanoscience

## Abstract

Plants have complex internal signaling pathways to quickly adjust to environmental changes and harvest energy from the environment. Facing the growing population, there is an urgent need for plant transformation and precise monitoring of plant growth to improve crop yields. Nanotechnology, an interdisciplinary research field, has recently been boosting plant yields and meeting global energy needs. In this context, a new field, “plant nanoscience,” which describes the interaction between plants and nanotechnology, emerges as the times require. Nanosensors, nanofertilizers, nanopesticides, and nano‐plant genetic engineering are of great help in increasing crop yields. Nanogenerators are helping to develop the potential of plants in the field of energy harvesting. Furthermore, the uptake and internalization of nanomaterials in plants and the possible effects are also worthy of attention. In this review, a forward‐looking perspective on the plant nanoscience is presented and feasible solutions for future food shortages and energy crises are provided.

## Graphical abstract

This review provides the first detailed introduction of plant nanoscience, including nanosensors, nanofertilizers, nanopesticides, nanomaterials‐mediated gene transformation, nanogenerators, and nanotoxicology. The interaction between nanomaterials and plants, as well as, the advanced application fields of nanotechnology in plants are discussed deeply to better manage the growth of plants and offer guidance for the future research and practical applications.

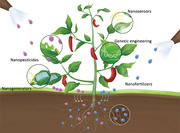

## 1 Introduction

The global population, currently 7.7 billion, is expected to rise to 9.8 billion by 2050.^[^ ^1^ ^]^ To meet the food demand of the ever‐increasing population, it is urgent to ensure the sustainable growth of crop yields.^[^ ^2^ ^]^ However, agriculture production is facing a series of environmental and biological challenges.^[^ ^3^ ^]^ Therefore, it makes sense to precisely manage the limited resources and increase crop yields in a sustainable way.^[^ ^4^ ^]^ The green revolution, which began in the 1960s, achieved the necessary increase in crop yields with low land use. However, as agricultural development becomes increasingly inefficient and unsustainable, this revolution came to an end. Instead, the second green revolution gradually emerged. This new revolution, aimed at meeting future growing food needs and achieving sustainable development, is driven by new technologies and materials.^[^ ^5^ ^]^ Among them, nanotechnology, a fast‐growing field for processing materials at the nanoscale, stands out.^[^ ^3^, ^6^, ^7^, ^8^, ^9^, ^10^ ^]^ With the gradual penetration of nanotechnology in the field of plants, the combination of plants and nanotechnology may give birth to a new field of research, which can be called “plant nanoscience” here.

Recently, nanotechnology‐enabled sensors (namely nanosensors) play a unique role in agriculture. Nanosensors have their own unique nano‐interface, which can specially recognize the received signals and amplify the signals, providing highly sensitive, stable, and reproducible detection even at the single molecule level.^[^ ^11^, ^12^, ^13^, ^14^ ^]^ They can directly transfer signals released by plants into digital information on the connected electronic devices, which are time‐saving and efficient compared to traditional detection techniques.^[^ ^4^, ^15^, ^16^, ^17^, ^18^, ^19^ ^]^ Nanosensors facilitate the transition from traditional macro‐scale plant monitoring to automated micro‐scale monitoring and information acquisition, which helps to accurately mange the process of plant growth.

In addition to the real‐time monitoring of plant to achieve better agricultural production, attention should also be paid to the provision of nutrients and pest management during plant growth. However, the commonly used instantly soluble fertilizers not only have low utilization efficiency (<30%) but also cause environmental pollutions.^[^ ^20^ ^]^ Furthermore, large amounts of chemical pesticides are lost during the application, causing the contamination of ecosystem.^[^ ^21^, ^22^ ^]^ Therefore, it is important to develop new strategies to overcome the above problems. Nanotechnology‐enabled fertilizers (namely nanofertilizers) and pesticides (namely nanopesticides) have the properties of high efficiency, durability, and biocompatibility in the application process.^[^ ^23^, ^24^, ^25^, ^26^ ^]^ They are excellent substitutes for traditional agrochemicals and can effectively protect plants from nutritional deficiencies and pests.^[^ ^27^, ^28^ ^]^

Although nanofertilizers and nanopesticides help to improve crop yields and quality, it is also necessary to solve these problems fundamentally, such as planting high‐yield plants that are naturally resistant to pests. Recently, genetic enhancement of plants has been widely employed to increase the yields and provide plants with robust resistant to biotic and abiotic stresses.^[^ ^29^, ^30^, ^31^ ^]^ Plant gene transformation usually involves regulating the existing genetic material or delivering required genes to the host cells, thereby imparting new characteristics to plants.^[^ ^32^ ^]^ Extensive studies have confirmed that nanomaterials can delivery biomolecules into animal cells, but their application in plants lags behind, mainly due to the cell wall recalcitrance to nanomaterials internalization.^[^ ^10^, ^33^, ^34^, ^35^, ^36^, ^37^ ^]^ However, some recent breakthroughs have accelerated the research process of using nanomaterials as delivery vehicles (nanocarriers) in plants.^[^ ^38^, ^39^, ^40^, ^41^, ^42^ ^]^ Nanocarriers represent a promising way for plant genetic engineering due to their specific properties like large surface area for chemical modification and biocompatibility, protecting the loaded biomolecules from the threat of cell metabolism and degradation.^[^ ^43^ ^]^

As can be seen from the above comments, nanotechnology has made it possible to accurately manage plant growth processes. But for some nanotechnologies, especially nanosensors, energy supply is still a big problem because of the ongoing energy crisis. Plants are widely distributed in the environment, and it is of great interest to utilize plants to obtain sustainable energy. Besides the most common solar energy, plants can also capture the ubiquitous mechanical energy in the environment.^[^ ^44^ ^]^ If the mechanical energy can be converted into electrical energy for use in some way, it will be very helpful to alleviate the energy crisis. The triboelectric nanogenerator (TENG), which can generate electricity based on the coupling effect of triboelectrification and electrostatic induction, has attracted great attention for low‐cost, high efficiency, high power density, and simple structure.^[^ ^45^, ^46^ ^]^ It is worth noting that plants have participated in the construction of TENG to capture the abundant mechanical energy in the environment.^[^ ^44^, ^47^, ^48^, ^49^ ^]^ Therefore, the combination between TENG and plant may provide new ideas for the new and green energy.

Although nanotechnologies bring us a lot of convenience, the potential toxicity from nanomaterials may cause risks for living organism and produce unknown byproducts in the environment. The study of nanotoxicology help us better understand the interactions between nanomaterials and living organism.^[^ ^50^, ^51^ ^]^ The size, surface area, morphology, concentration, and other characteristics of nanomaterials may affect the physiological processes of plants. We need to explore the uptake and transformation of nanomaterials in plants, thereby designing more efficient nanomaterials to keep the hazards of the substances at a minimum.

**Figure 1:**
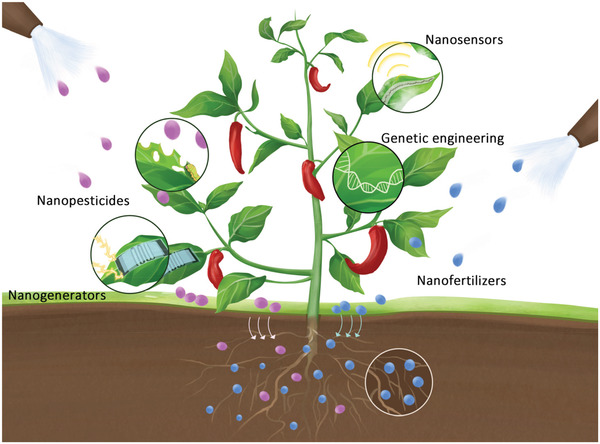
The plant nanoscience.

This review summarizes the wide applications of nanotechnology in plants in recent years around the topic of plant nanoscience, including nanosensors, nanofertilizers, nanopesticides, nanocarriers, nanogenerators, and nanotoxicology (**Figure** **1**). In light of the emerging field of plant nanoscience, we emphasize the importance to view all proposed agri‐tech solutions from a systematic perspective to ensure that they are sustainable, safe, and able to cope with future food supply and energy crisis.

## 2 Nanosensors for Plant Monitoring

Plants have continuous gas and fluid exchange with the environment through their leaves and roots.^[^ ^52^ ^]^ The continuous changes of the environment directly reflect the growth status of plants, and plants can also reflect the fluctuations of their surroundings. Traditional plant phenotypic technologies, such as “3S” technologies (remote sensing (RS), geography information systems, and global positioning systems),^[^ ^15^, ^16^, ^17^ ^]^ spectroscopy,^[^ ^18^ ^]^ machine vision and digital image capture/analysis,^[^ ^19^ ^]^ etc. These technologies are suitable for the perception of plant phenotypic information at macro‐scale such as chlorophyll and nitrogen content, canopy information, leaf area index, and pest infestation. However, critical information such as the information exchange between plants, response to environmental stress, and internal physiological signal changes at the micro‐nano scale cannot be obtained by these technologies.

**Figure 2:**
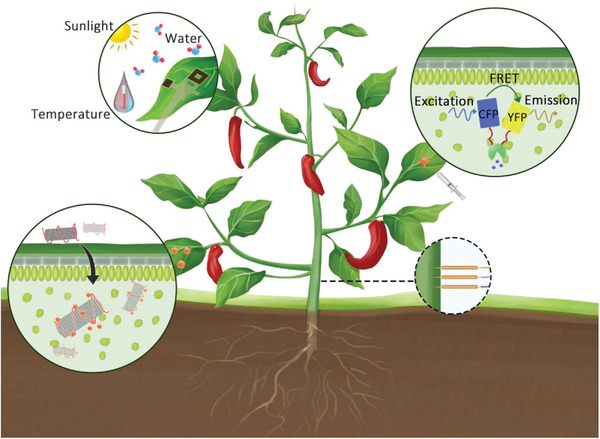
Nanosensors for plant monitoring.

Nanotechnology, one of the most important research hotspots in the world, has great potential to enable plants to communicate with and actuate electronic devices, helping people to intuitively understand the physiological state of the plant and the dynamic changes of its surroundings.^[^ ^4^ ^]^ Facing with the challenges of current agricultural development, nanosensors have achieved accurate, real‐time, and high spatiotemporal resolution monitoring of individual plant at the micro‐scale, and translate these signaling molecules generated by plant via optical, wireless or electrical signals, thus helping people to better control all aspects of agricultural production. As shown in **Figure** **2**, these smart nanosensors monitor plants and their surroundings primarily in vitro and in vivo, and their applications will be discussed in more details below.

### 2.1 In Vitro Monitoring

#### 2.1.1 Plant Growth

When observing the process of the plant growth, the most intuitive change is the continuous change of the external shape as plant grows from the seedling. Therefore, various methods have been used for measuring the changes of circumference of plants, including manual instruments like measuring tapes or calipers, and fully automated systems like strain gauges,^[^ ^53^ ^]^ linear potentiometers,^[^ ^54^ ^]^ etc. But they usually have low detection accuracy, low spatial resolution of nanometer‐scale precision, and limited location, requiring regular adjustments of the frame during long‐term measurements.^[^ ^55^ ^]^ Strain sensors are devices that convert mechanical deformations into the changes of electrical characteristics like resistance.^[^ ^2^ ^]^ And with the current development of flexible and stretchable electronics, wearable strain nanosensors have emerged with the ability of real‐time and continuous monitoring. Although most of them are applied to monitor human motion rather than plant growth.^[^ ^56^, ^57^, ^58^ ^]^ Given their unique advantages into account, it is highly desirable to make wearable strain nanosensors with high sensitivity and wide sensing range for in situ and real‐time measuring of plant growth.

**Figure 3:**
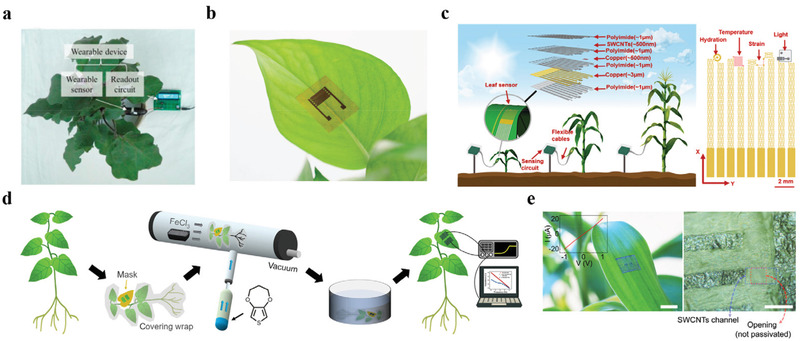
Flexible and wearable nanosensors for in vitro plant growth monitoring. a) Images of the all‐in‐one wearable device for plant growth measurement. Reproduced with permission.^[^ ^2^ ^]^ Copyright 2019, Elsevier Ltd. b) Photo of the GO‐based flexible humidity nanosensor that attached to the surface of a leaf. Reproduced with permission.^[^ ^67^ ^]^ Copyright 2020, Elsevier Ltd. c) Schematic diagram of the multifunctional stretchable sensor on a leaf and the top view of the leaf sensor. Reproduced with permission.^[^ ^68^ ^]^ Copyright 2019, American Chemical Society. d) The process of the vapor coating living plants with functional polymer films. Reproduced with permission.^[^ ^69^ ^]^ Copyright 2019, American Association for the Advancement of Science. e) Photo (left), an optical microscope image (right), and the *I*–*V* characteristic (inset) of SWCNTs/graphite arrays laminated onto surface of a live leaf. Reproduced with permission.^[^ ^72^ ^]^ Copyright 2014, American Chemical Society.

The most distinctive advantage of the wearable strain nanosensor is that it can be closely attached to the surface of the plant. With the growth of the plant, a certain tension will be exerted on the nanosensor, which will make the nanosensor stretch accordingly, leading to changes in the output electrical signals. Based on this, the adhesion, flexibility, sensitivity, transparency, biocompatibility, etc., are the evaluation indexes of these wearable strain nanosensors. Especially for plants, it is necessary to consider whether can reflect the micro‐changes of plant growth at micro‐nano scale, whether they can firmly adhere to the surface of plants with abundant microstructures, whether they pose a threat to plant growth, and whether the real‐time in situ monitoring can be achieved. In existing researches, our group fabricated a flexible strain nanosensor by writing the chitosan‐based conductive ink, which was synthesized by mixing graphite nanopowder and chitosan solution, directly on cucumbers (*Cucumis sativus* L.).^[^ ^59^ ^]^ Results showed that within strain from 1% to 8%, the fabricated nanosensor had a gauge factor (GF) value of 64 and can be stretched up to 60%, showing the ability to monitor plant growth. Later, the performance of the nanosensor was improved by introducing a carbon nanotube (CNT) ink, and successfully monitored the growth of another two plants (*Solanum melongena* L. and *Cucurbita pepo*).^[^ ^2^ ^]^ This wearable strain nanosensor was directly drawn on a disposable latex glove (**Figure** **3**a). And the synergistic reinforcement between graphite and CNT significantly improved the mechanical stability and stretchability of the nanosensor, which could respond to all strain loads from 0 to 150%, and the GF was 352 at 150% strain. This nanosensor exhibited higher spatiotemporal resolutions and sensitivity, demonstrating the two plants had a rhythmic growth pattern. Although neither of the wearable strain nanosensors have been used to continuously monitor plant growth over a long period of time, nor to validate their performance in the field. They still open up a new way for the development of wearable nanodevices in the field of plant nanoscience.

#### 2.1.2 Environmental Factors

Plants still live in a constantly changing environment, which is sometimes not conducive to their growth.^[^ ^60^ ^]^ Humidity level fluctuates with changes in surrounding temperature, and the opening and closing of leaf stomata in the process of photosynthesis is directly related to the change of temperature and humidity. Moreover, plants are also threatened by pests and diseases during their growth. All in all, these fluctuating environmental factors will more or less cause a series of reactions in the cells, and ultimately affect the physiological processes of plants. Plants often exhibit unique and complex responses to environmental changes, utilizing intercellular communication to integrate signals from different tissues and organs.^[^ ^61^, ^62^ ^]^ These signals usually consist of controlled production of plant hormones and rapidly spreading potential waves of cell membranes polarization, measuring these signals may provide information about fluctuating environmental factors.^[^ ^52^, ^63^ ^]^ Therefore, the use of nanosensors to communicate with plants is helpful to understand their growth mechanisms, and real‐time tracking these signals is of great significance to increase plant yields.

Of all the environmental factors, water is the most important for plant growth, because it controls the photosynthesis and transpiration process.^[^ ^64^ ^]^ Compared with the sensors arranged in the soil or among plants, the wearable nanosensors directly attached to plants can more intuitively reflect the small changes in the water status of plants themselves, especially in the early detection. Based on this, researchers have carried out various researches. Oren et al. utilized graphene‐based on‐tape nanosensors that patterned onto the back surfaces of the leaves of maize plants to detect the changes of relative humidity (RH) on the surface of the leaves.^[^ ^65^ ^]^ Upon irrigation, the RH of the leaves change, which is directly expressed as the change in the resistance of the nanosensor. Im et al. fabricated a flexible capacitive humidity nanosensor by depositing titanium (Ti) and gold (Au) on the polyimide (PI) film to form an interdigitated electrode.^[^ ^66^ ^]^ The transpiration of the stoma was monitored by the nanosensor that converting plants response variation into electrical signals, which were further sent to a mobile device wirelessly in real time. Although the performance of the mentioned nanosensors are good, it will be a very meaningful innovation if they are further combined with wireless devices to realize the real‐time transmission and analysis of monitoring data. And their fabrication procedures are very complicated and time‐consuming. The long‐term stability also has not been verified. Based on this, our group proposed a convenient and effective way for the quantity production of wearable capacitive‐type humidity nanosensors by using laser direct writing technology.^[^ ^67^ ^]^ A laser‐induced graphene interdigital electrode (LIG‐IDE) was fabricated on the PI film, and graphene oxide (GO) aqueous solution was further drop‐casted on the surface of LIG‐IDE to act as the humidity sensing material. This GO‐based flexible humidity nanosensor could be well attached to the surface of plant leaves without disturbing their normal growth, and it realized the real‐time and long‐term tracking of plant transpiration (Figure 3b). However, if the nanosensor is further combined with wireless devices to form an integrated system, and corresponding field trials are carried out, it could be more efficient for plant monitoring.

In addition to the monitoring of single environmental factors like humidity, the development of nanosensors for multi‐factor monitoring is also very necessary. Such innovative design is more time‐saving and efficient. Nassar et al. fabricated an autonomous wearable nanosensor for localized microclimate monitoring by patterning interdigitated capacitive structures of Ti/Au on PI film, and further transferred this multisensory structures onto the polydimethylsiloxane layer.^[^ ^60^ ^]^ This device could obtain real‐time humidity and temperature levels through the continuous monitoring on the surface of plant leaves. Furthermore, the physiological changes of plant leaves are closely related to the localized microclimate. By monitoring the leaf physiology and localized microclimate simultaneously, we can establish a link between them, which is helpful to better understand the physiological changes of plants. Therefore, Zhao and coworkers developed a multifunctional leaf‐mounted nanosensor, including a copper (Cu) film acted as hydration sensing element, a Ti/Cu film acted as temperature sensing element, single‐walled carbon nanotubes (SWCNTs) acted as strain sensing element, and SU‐8 encapsulated phototransistor acted as light sensing element.^[^ ^68^ ^]^ This multifunctional nanosensor could monitor leaf physiological and different environmental conditions continuously, and could extend with the growth of the attached leaves without disturbing the normal growth of the plant (Figure 3c).

These aforementioned nanosensors are all built on a flexible substrate to realize the detection process. Whether they can be firmly attached to the surface of the plant during long‐term use remain to be further verified. Moreover, the biocompatibility of the substrate material also needs to be considered. Based on this, Kim and coworkers demonstrated a novel concept of using biocompatible conducting polymer films as a nanosensor by directly printing the films onto living plants, which could detect deep tissue damages caused by dehydration and ultraviolet‐A exposure through non‐invasive bioimpedance spectroscopy (Figure 3d).^[^ ^69^ ^]^ They found that the vapor‐printed nanosensor could be used as durable diagnostic handles for detecting the sources of plant stress like drought and photodamage. This study provides a good idea for the construction of nanosensors for environmental factors monitoring in the future, which will play significant roles in plant biology and precision agriculture. However, as mentioned above, these multifunctional nanosensors also should be combined with wireless devices to form integrated systems. And, they also need to conduct long‐term and field trials to verify their performance.

The release of VOCs is an important way for phytochemical resistance mechanisms. They act as secondary metabolites and can be used for plant communication with other organisms and play a crucial role in interacting with biotic and abiotic stresses.^[^ ^70^ ^]^ Wearable nanosensors have been proven to be capable of monitoring environmental factors such as humidity, temperature, and illumination. Thus, taking advantage of the unique properties of them, they also have the potential to detect VOCs released by plants by using different sensitive materials to form different nano‐interfaces. Esser et al. utilized a mixture of SWCNTs and copper(I) complexes to fabricate a reversible chemo‐resistive nanosensor, which was able to detect ethylene at sub‐ppm concentrations from various fruits.^[^ ^71^ ^]^ The binding of ethylene to the SWCNTs/copper(I) complexes reversibly increases the resistance of SWCNTs. However, this nanosensor is fabricated directly on a rigid substrate, which is not convenient for their application in plants. Thus, in another study, Lee et al. fabricated wearable electronic devices in situ based on the integrated arrays of SWCNTs transistors with graphitic electrodes and interconnects.^[^ ^72^ ^]^ These devices could realize real‐time monitoring of toxic gases (dimethyl methylphosphonate vapor) by transferring and laminating directly on plant leaves (Figure 3e).

In addition to the aforementioned electrical signals that reflect the variation of VOCs content, some more intuitive colorimetric detection methods can also be used. Li and coworkers fabricated a low‐cost and integrated smartphone‐based VOC fingerprinting platform for early and non‐invasive detection of gaseous (*E*)‐2‐hexenal at sub‐ppm level (one of the main VOC markers emitted from tomatoes that infected by *Phytophthora infestans*) by using a paper‐based colorimetric sensor array.^[^ ^73^ ^]^ In this VOC‐sensor array, 5 kinds of cysteine‐functionalized Au nanomaterials were used as plasmonic aggregative colorants and five conventional organic dyes were used for specific detection of (*E*)‐2‐hexenal, where the process of “signals generation‐colour changes” could be quantified by smartphones within 20 min. It also helped to diagnose tomato late blight as early as 2 days, and the diagnostic accuracy of the leaf samples collected in the laboratory or from the field was more than 95%. Inspired by this study, future nanosensors should have early high‐precision detection capabilities, and can be integrated with wireless devices to transmit monitoring data to mobile devices in real time. Moreover, if they can realize in situ monitoring in a non‐destructive and biocompatible form, it will be more conducive to their application in plants.

#### 2.1.3 Soil Conditions

Soil is the foundation of plant growth, providing a constant supply of nutrients for plant growth. Many important ecological processes in nature are carried out in the soil, such as the transformation of pollutants. The indicators for soil quality evaluation include water content, pH, organic matter content, pollutants content, etc. Among them, the detection of various pollutants is very important, because they not only inhibit plant growth and cause great loss of crop yield, but also increase the absorption of pollutants by crops, thereby contaminating the food chain. The general steps for determining the pollutant content in soil usually include field sampling, plant tissue digestion, extraction, sample purification, etc., and the methods used are mostly mass spectrometry, chromatography, electrochemical detection, etc.^[^ ^74^, ^75^, ^76^, ^77^ ^]^ The complicated and time‐consuming sampling process requires expensive instruments and professional operations. Moreover, the on‐site and real‐time monitoring cannot be achieved.

Based on the above considerations, a reliable technology is needed to achieve rapid, on‐site, and real‐time assessment of pollutants in the soil. In a study, researchers designed a new type of plant nanobionic sensor for selective monitoring the arsenic content in the soil.^[^ ^78^ ^]^ Arsenic and its compounds are serious threats to humans and ecosystems. Here, living plants are used to interface with specially designed nanomaterials as self‐powered detectors. The authors designed SWCNTs‐based near infrared (NIR) fluorescent nanosensors, which are embedded in plant tissues with no harmful effects, providing a non‐destructive method to monitor the internal dynamics of plants absorbing arsenic from the soil. This plant nanobionic approach of integrating optical nanosensors inside living plants represents a great improvement compared with traditional methods. In the future, this new type of nanobionic sensor will play an important role in soil quality monitoring, and provide ideas for the research of other nanosensors for soil condition detection. The effective assessment of soil quality will be achieved by combining portable and inexpensive electronic equipment with living plants, which is a real‐time and on‐site method.

### 2.2 In Vivo Monitoring

Plants possess complex signaling pathways to perceive changes in the surrounding environment. And to cope with various environmental stresses, plants reprogram their cellular transcription mechanism and metabolic responses through these signaling pathways to enhance their endurance under different stresses.^[^ ^52^, ^79^ ^]^ The signaling molecules usually can be divided into intercellular and intracellular signaling molecules according to their scope of action, and they can spread throughout the plant within seconds to trigger systemic signaling mechanisms.^[^ ^52^, ^80^, ^81^ ^]^ Revealing the dynamic changes of various signaling molecules under the external environment, which is a key step in understanding how plants coordinate the activities of many cells into a multicellular whole in a dynamic environment.

Generally, the use of microelectrode‐based nanosensors to realize in situ detection is considered as non‐destructive, and their fast response time make them highly powerful tools for studying the in vivo physiological changes of plants. For another type, the genetically encoded nanosensors, which have been developed from fluorescent proteins, are able to report the changes in intracellular molecular concentration, presence and activity of proteins, several key plant hormones, metabolites, and ion dynamics through the changes in fluorescence intensity, fluorescence resonance energy transfer (FRET), or bioluminescence resonance energy transfer.^[^ ^4^, ^82^, ^83^ ^]^ Although their application may be limited to specific genetically modified species and are still in the early stage of research, this situation has been alleviated with the assistance of nanomaterials. As an alternative, optical nanosensors can be widely used in plants that are currently not suitable for genetic modification.^[^ ^52^ ^]^ Nanomaterials like SWCNTs and quantum dots (QDs), with unique optical and electronic properties, have advantages in imaging plant signaling molecules in vivo. Various delivery methods for these optical nanosensors can be performed in laboratories, and in field experiments such as needleless syringe infusion through plant leaves, topical delivery, and vacuum infiltration.^[^ ^4^ ^]^ These aforementioned in vivo nanosensors are powerful tools for monitoring changes of signaling molecules in plants, and their specific applications will be described in detail below.

#### 2.2.1 Metal Ions

Metal ions such as calcium (Ca^2+^), potassium (K^+^), sodium (Na^+^), and magnesium (Mg^2+^) ions are important signaling molecules in plants. Different external stimuli can cause metal ions transient increases, waves, and oscillations, which reflects the physiological changes of plants.^[^ ^81^ ^]^ Moreover, monitoring changes in the concentration of intracellular metal ion concentrations is also important for diagnosing plant nutritional status.^[^ ^84^ ^]^ Therefore, it is necessary to conduct studies on the dynamic changes of metal ions in plants.

The most used technology for metal ions detecting in plants mainly rely on the construction of ion‐selective microelectrode.^[^ ^85^, ^86^, ^87^ ^]^ This technology can realize the noninvasively measurement of metal ions fluxes in plant root elongation zone or suspension cell. However, the detection process is complex and requires high precision of operation. Therefore, it is necessary to develop a simple and fast method. For this, Janni et al. prepared an in vivo organic electrochemical transistor sensor based on poly(3,4‐ethylenedioxythiophene):poly(styrenesulfonate) functionalized textile thread, termed as bioristor, for measuring changes of different metal ions concentrations (Na^+^, K^+^, Ca^2+^, and Mg^2+^) in the sap of drought‐stressed tomato plants.^[^ ^88^ ^]^ The device was integrated with the stem and realized the continuously monitoring of the plant's physiological status, showing that the concentration of metal ions gradually increases within 30 h without water supply. The device can be used as a useful tool for breeding drought‐resistant tomato cultivars. However, this device is directly inserted into the stem of the plant, so the stability and biocompatibility during long‐term use, and the interaction mechanism between the nanomaterials used and the plant system need to be further investigated. In addition, the data is still transmitted by wire, which is very inconvenient for the long‐term use, thus it also needs to be integrated with wireless devices in the future.

#### 2.2.2 Reactive Oxygen Species

Reactive oxygen species (ROS) play a variety of signal transduction roles in different organisms, which mainly includes superoxide anion (O^2−^), hydrogen peroxide (H_2_O_2_), carboxyl radical (—OH), and nitric oxide (NO), etc. From the toxic byproducts of aerobic metabolism, ROS have become important roles in the complex signaling network of cells.^[^ ^89^ ^]^ The release of ROS is a quite universal and fast defense mechanism in plants, which is known to be associated with various plant pathogen interactions or environmental stresses. ROS have also been acknowledged as important regulators of plant growth, having great influences in the process of transducing hormone signals and regulating the structure of cell wall polymers.^[^ ^90^, ^91^ ^]^ Therefore, it is important to study the production of ROS and their activities between compartments in the process of plant growth.

**Figure 4:**
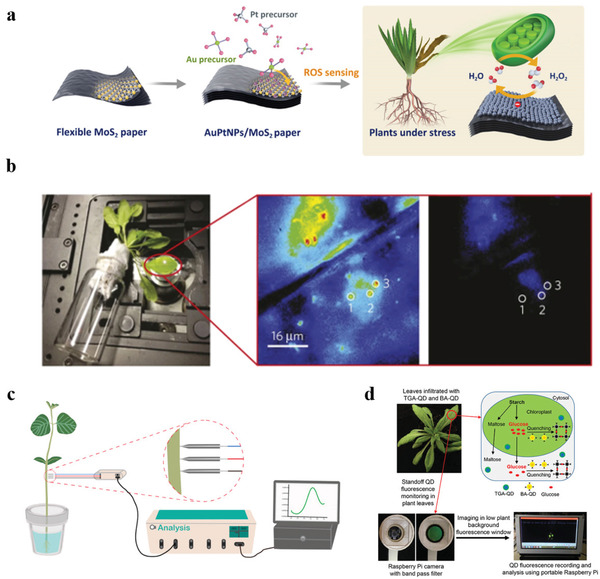
Nanosensors for in vivo plant growth monitoring. a) Schematic diagram of the spontaneous growth of AuPt NPs on flexible MoS_2_ paper and the application in the determination of H_2_O_2_. Reproduced with permission.^[^ ^94^ ^]^ Copyright 2020, Elsevier Ltd. b) In vivo plant sensing set‐up where a leaf infiltrated with SWCNTs was excited by a 785 nm epifluorescence microscope (left). The 20‐fold magnification view of SWCNTs inside a leaf before and after the addition of 20 µL dissolved NO solution, where three SWCNTs regions were circled (right). Reproduced with permission.^[^ ^98^ ^]^ Copyright 2014, Nature Publishing Group. c) Photo of the stainless steel microelectrode to monitor IAA in the stem of soybean. Reproduced with permission.^[^ ^111^ ^]^ Copyright 2019, Elsevier Ltd. d) Schematic diagram of in vivo glucose sensing and standoff imaging by QD fluorescent probe through a Raspberry Pi camera. Reproduced with permission.^[^ ^128^ ^]^ Copyright 2018, American Chemical Society.

Various technologies have been used for monitoring changes of H_2_O_2_ in plant leaves, and the mostly commonly used is fluorescent probe. However, the traditional fluorescent probe technology is time‐consuming and needs to separate plant samples. Hence, before the emergence of better H_2_O_2_ fluorescence detection technologies in plants, electrochemical methods with the advantages of simplicity, low‐cost, and high sensitivity have been widely used in the study of plant biochemical processes. For example, Ren and coworkers utilized hemoglobin (Hb) and SWCNTs modified carbon fiber ultramicroelectrode (Hb/SWCNTs/CFUME) for electrochemically in vivo monitoring the changes of H_2_O_2_ level in aloe leaves under salt stress.^[^ ^92^ ^]^ The Hb/SWCNTs/CFUME was inserted into the center of the leaf and realized the amperometric in vivo monitoring of H_2_O_2_ due to the catalytic effect of Hb on the reduction of H_2_O_2_. In another study, Lima et al. realized the in situ monitoring of H_2_O_2_ produced in *A. tequilana* leaves after inoculating with bacteria by using a platinum (Pt) disc microelectrode.^[^ ^93^ ^]^ The electrochemical results revealed the relationship between the inoculated leaves and the production of H_2_O_2_. And our group also fabricated a high‐performance ROS electrochemical nanosensor (**Figure** **4**a).^[^ ^94^ ^]^ Ultrathin metallic molybdenum disulfide (MoS_2_) nanosheets were used to fabricate freestanding paper electrode, and noble metal alloy NPs (AuPt NPs) with excellent catalytic activity were spontaneously coated on the electrode. The flexible nanosensor exhibited great mechanical properties and excellent performance in H_2_O_2_ monitoring in plant extract of aloes with the advantages of convenient, prominent selectivity and stability, and wide linear range. These electrochemical nanosensors allow in situ monitoring with high selectivity and stability, eliminating tedious data processing. However, most of them are investigated in the laboratory, and the biocompatibility of these nanosensors has not been rigorously verified. Moreover, it is necessary to develop electrochemical nanosensors that can realize in situ monitoring on living plants in the future and can be integrated with wireless devices, achieving a highly integrated, miniaturized, and portable monitoring system.

Although electrochemical‐based detection methods are simple and have high sensitivity, in vivo optical nanosensors are more advantageous for non‐destructive and real‐time monitoring of specific molecules, especially when we need to fully understand the dynamic changes of specific physiological processes. Especially for SWCNTs‐based in vivo optical nanosensors, which have electronic structure characteristics from metals to semiconductors with a certain energy band gap, thereby electrons transition between these energy bands to realize light absorption and emission. And they usually fluoresce in the NIR region (800–1600 nm), where chloroplast autofluorescence is minimal. Moreover, SWCNTs can be chemically functionalized, making them high sensitivity and specificity to targets. Therefore, they have unique advantages in enabling the detection of analytes with high spatiotemporal resolution down to the single‐molecule level and millisecond timescale.^[^ ^4^, ^95^, ^96^ ^]^ Giraldo et al. first delivered 6,5 ss(AT)_15_‐SWCNTs to chloroplasts of *Arabidopsis thaliana* leaves by infiltrating through the leaf lamina, realizing the in vivo detection of NO, which is a key signaling molecule within chloroplasts (Figure 4b).^[^ ^97^, ^98^ ^]^ Results showed that when there was dissolved NO in vivo, the SWCNTs chiralities with emission peaks above 1100 nm exhibited a strong quenching in NIR fluorescence, exhibiting a multi‐chirality property. Therefore, the group referred to the leaves embedded with SWCNTs as nanobionic leaves, which imparted the plant leaves with novel sensing capabilities to realize biochemical detection of endogenous signaling molecules and exogenous environmental compounds. This study brought new research fields for the combination of nanotechnology and plant biology, and this, in vivo optical nanosensors can also be extended to detect other plant signaling molecules and exogenous compounds.

However, for the fluorescence responses of multi‐chirality SWCNTs with multiple NIR emission peaks, the signals observed by optical nanosensors were usually limited, and the detection accuracy may be reduced in the presence of other interfering substances.^[^ ^98^, ^99^, ^100^ ^]^ Moreover, when a nanosensor is embedded into a biological sample that is complex and constantly changing, it is often difficult for them to show high selectivity in the presence of abundant interfering molecules. To solve this problem, the same group developed the ratiometric fluorescent nanosensors for in vivo sensing of H_2_O_2_ and NO in plant tissues.^[^ ^101^ ^]^ For the H_2_O_2_ detection, the 7,6 ss(GT)_15_‐SWCNTs (emission = 1131 nm) were quenched in the presence of H_2_O_2_ and the 6,5 ss(AT)_15_‐SWCNTs (emission = 984 nm) were invariant to H_2_O_2_. For the NO detection, 7,6 ss(GT)_15_‐SWCNTs (emission = 1135 nm) were quenched in the presence of NO and the 6,5 polyvinyl alcohol‐SWCNTs (6,5 PVA‐SWCNTs) (emission = 1004 nm) were invariant to NO. Results showed that, in each case, there was a pair of SWCNTs fluorescence emitters, where only one responded to the target analyte and the other served as a reference signal. Such ratiometric fluorescent nanosensors has high selectivity for the target to be detected, which has advantages for specific detection in complex biological samples.

As the generation and accumulation of ROS like H_2_O_2_ is a sign of plant responses to stress, which often includes high light, high temperature, salinity, wounding, and pathogen infection.^[^ ^89^, ^102^, ^103^ ^]^ Therefore, the group further how the ROS fluctuates when plants are under environmental stresses. They utilized the SWCNTs‐based ratiometric fluorescent nanosensors to continuously monitor the H_2_O_2_ signaling waveform induced by wounding in different plant cultivars and genetic variants.^[^ ^104^ ^]^ Results showed that the H_2_O_2_ wave follows a logistic waveform, and the H_2_O_2_, electrical, and Ca^2+^ signaling pathways interacted to regulate the defense response of plants after wounding. In another study, Wu el al. developed a H_2_O_2_ fluorescent nanosensor based on SWCNTs functionalized with a deoxyribonucleic acid (DNA) aptamer that bound to hemin (HeAptDNA‐SWCNTs), allowing the remote in vivo monitoring of H_2_O_2_ within the plant physiological range (10–100 µM).^[^ ^105^ ^]^ Results showed that this nanosensor could realize the real‐time monitoring of several stresses, for example, UV‐B light, high light, and pathogen‐related peptide through the change of H_2_O_2_.

These aforementioned in vivo optical nanosensors provide real‐time, efficient, and highly selective detection results. With the deepening of future researches, these nanosensors should be applied to various plant species rather than limited to model plants such as *A. thaliana*. And these nanosensors are still unable to measure the content changes of multiple substances at the same time, except for the most common H_2_O_2_ and NO. To detect the content of multiple substances, there needs to be certain differences between the fluorescence wavelengths of the nanomaterials. The nanomaterials currently used are still limited to SWCNTs, thereby the simultaneous detection of multiple substances is a challenge. Moreover, when nanomaterials like SWCNTs are used for optical sensing in plants, whether they have long‐term stability and whether they may pose threats to plants still need to be further investigated. Only by thoroughly studying the interaction mechanism between them and plants can the in vivo optical detection technology develop better. Last but not least, the current detection process for these optical nanosensors is done in a dark room. In field trials, sunlight will interfere with the fluorescence of the nanosensors, making them only suitable for researches in the laboratory.

#### 2.2.3 Plant Hormones

Plants have various hormones including auxins, gibberellins, abscisic acid, cytokinins, salicylic acid, ethylene, and peptide hormones, etc. They are small organic molecules or active substances, which are induced by plant cells upon receiving specific environmental signals and can regulate plant physiological responses at very low concentrations. Thus, they play important roles in the process of plants adapting to various environmental stresses.^[^ ^61^ ^]^ Various technologies has been used for detecting plant hormones, such as high‐performance liquid chromatography,^[^ ^106^ ^]^ gas chromatography‐mass spectrometry,^[^ ^107^ ^]^ fluorescence spectrometry,^[^ ^108^ ^]^ and capillary electrophoresis (CE),^[^ ^109^ ^]^ etc. But they can only be used in vitro, where plant samples need to undergo complex pretreatment and the procedure is time‐consuming. For in vivo detection, Hu et al. utilized Pt nanoflowers/reduced graphene oxide (rGO) modified Pt microelectrode to detect salicylic acid in plants.^[^ ^110^ ^]^ The authors punched the stem of sunflower seedlings with a puncture needle for the insertion of microelectrodes, thereby realizing the highly sensitive and selective detection of salicylic acid in plants under different salt stresses, where the limit of detection (LOD) is 48.11 pM. Similar to this method, Li and coworkers prepared highly ordered nanopores on the surface of a stainless steel microelectrode.^[^ ^111^ ^]^ They further utilized popcorn‐like Au nanostructures, Pt NPs and rGO nanocomposite films, and polymerized safranine T film to modify the electrode for the in vivo detection of indole‐3‐acetic acid (IAA) (Figure 4c). The microsensor realized the detection of IAA of soybean seedling stems under salt stress with high selectivity and sensitivity, and the LOD was as low as 43 pg L^−1^. These electrochemical nanosensors are measured by directly inserting microelectrodes into plants, the long‐term stability and biocompatibility have not been thoroughly investigated. Moreover, the whole detection process is conducted on the electrochemical workstation, and no field trial is carried out. Therefore, further investigations are needed to make these nanosensors work stably and resist the interference of environmental changes.

Optical nanosensors can also be used to detect plant hormones in vivo. QDs are promising nanomaterials with unique properties of bright fluorescence, broad excitation spectrum, tunable emissions, and good photostability, which have been widely used in biolabeling and bioimaging.^[^ ^112^, ^113^, ^114^ ^]^ And the conjugation of QDs with specific ligands such as aptamers helps to realize the specific imaging of target molecules. Based on this, Liu and coworkers developed a fluorescent aptasensor by using aptamer‐functionalized QDs (zinc ion (Zn^2+^) doped cadmium telluride) for the in vivo determination of tomato systemin (TomSys), which is a kind of peptide hormones in plants.^[^ ^115^ ^]^ Results showed that in the absence of TomSys, the fluorescence was quenched due to the absorption of aptamer‐functionalized QDs on the surface of GO nanosheets via the noncovalent interactions. While in the presence of TomSys, the fluorescence of QDs recovered due to the release of the aptamer‐functionalized QDs from the GO surface, and the aptamer was bound to TomSys. In addition, metal‐based fluorescent nanosensors have also been extensively developed for monitoring signaling molecules.^[^ ^116^ ^]^ Chen et al. developed a curcumin‐Cu ion (Cu^2+^)‐based fluorescent nanosensor for selectively and sensitively in vivo detecting of salicylic acid.^[^ ^117^ ^]^ Results showed that Cu^2+^ was first selected to bind the *β*‐diketone part of curcumin, resulting in the fluorescent “turn‐off” of curcumin due to the paramagnetic nature of Cu^2+^. Then, after the salicylic acid was introduced, the fluorescence “turn‐on” pattern was observed with high selectivity due to the affinity of salicylic acid toward Cu^2+^. Compared with the aforementioned electrochemical nanosensors, these fluorescence nanosensors exhibit higher selectivity and sensitivity, and the detection process can be observed more intuitively through the complex changes of fluorescence signal. However, as common problems of optical sensors mentioned above, their long‐term stability and biocompatibility in plants still need to be thoroughly investigated.

#### 2.2.4 Other Important Molecules

In addition to the aforementioned several important signaling molecules, the dynamic changes of other molecules in plants also play important roles in assessing stresses and determining the process of plant growth and development.^[^ ^118^ ^]^ These dynamic processes help to understand how plants perceive dynamic environmental changes and coordinate the activities of individual cells. Recently, there are increasing researchers have paid attention to exploring the metabolic process of plants, and conducting in‐depth researches on the real‐time monitoring of various metabolites of plants including adenosine triphosphate (ATP), lipid phosphatidic acid (PA), nicotinamide adenine dinucleotide phosphate (NADPH), and nicotinamide adenine dinucleotide (NADH) by using genetically encoded nanosensors.

Among them, ATP is an important product of photosynthesis and is used in the anabolism of chloroplasts, and the matching of the ATP:NADPH production and consumption in the chloroplast is a prerequisite for effective photosynthesis. Studies have demonstrated that chloroplasts can take up ATP from the cytoplasm at night to meet metabolic needs, while during the day or under stresses, chloroplasts can also export ATP to the cytoplasm.^[^ ^119^ ^]^ Therefore, it is important to figure out the dynamic changes of the in vivo ATP in these two compartments for better plant growth. Voon et al. utilized a MgATP^2−^‐specific FRET‐based genetically encoded nanosensor for the measurement of intracellular compartmentation of ATP in living plants.^[^ ^120^ ^]^ ATP is usually complexed as MgATP^2−^ in leaves. After imaging the expression of the fluorescent ATP protein in *A. thaliana* seedlings, the authors revealed that the concentration of MgATP^2−^ was higher in the cytosol than in the stroma of mature chloroplasts, which was because only immature chloroplasts needed to absorb cytoplasmic ATP for biosynthesis in the early stages of development. When the chloroplast is fully developed and able to maintain itself, it is necessary to down‐regulate the expression of ATP nucleotide transporter.

PA is an important raw material for the formation of glycerol‐phospholipids and triacylglycerol, and it acts as a significant signaling molecule that participates in regulating various fundamental plant cellular functions, especially in regulating the stomatal movement and drought or salt stress responses.^[^ ^121^, ^122^, ^123^ ^]^ Therefore, Li et al. developed a genetically encoded nanosensor based on FRET (PAleon) for the monitoring of the dynamics of PA at the plasma membrane.^[^ ^124^ ^]^ PAleon is a PA probe, which is sensitive to monitor the dynamic changes of PA in tissues when plants under abscisic acid or salt stresses. Results showed that, under the abscisic acid or salt stresses, PAleon reported a rapidly induced accumulation of PA in root tissues and guard cells without affecting the endogenous signaling system and the normal growth of plants.

NADPH and NADH are crucial energy molecules in living systems and NADH/NAD^+^ are a ubiquitous cellular redox couple, which are all closely related to the metabolic activities of plants. Most researches have conducted in vitro measurements by extracting these metabolites from tissues, which is time‐consuming and cannot realize the real‐time measurement of the instantaneous levels of these metabolites in different subcellular compartments. Therefore, Lim et al. developed two kinds of genetically encoded nanosensors (iNAP and SoNar) based on circularly permuted yellow fluorescent protein (YFP), realizing the dynamic monitoring of NADPH levels in NADPH pool and the NADH/NAD^+^ ratio in *A. thaliana* under light condition.^[^ ^125^ ^]^ Results showed that in the process of photorespiration, large amounts of NADH were produced in mitochondrias under the action of glycine decarboxylase and further oxidized in peroxisomes under the action of hydroxypyruvate reductase concurrently, where the changes of NADH/NAD^+^ ratio in the cytosolic reflected this phenomenon. These findings confirmed the complex interactions between chloroplasts and mitochondria during photosynthesis, helping to improve the efficiency of plant photosynthesis in the future. In another study, Steinbeck and coworkers studied the subcellular redox dynamics of NADH in *Arabidopsis* tissues based on the genetically encoded nanosensor by using a cyan fluorescent protein and a red fluorescent protein.^[^ ^126^ ^]^ It monitored changes in NADH/NAD^+^ in vivo, real‐time reflecting the effects of environmental changes (e.g., light, temperature, drought, and pest infestation) on plant metabolism. This study generated the first living Arabidopsis cytosol NADH redox atlas and observed the redox dynamics through the dark‐light transitions, respiratory inhibition, and changes of sugar supplement.

For other key plant signaling molecules like glucose, some studies have demonstrated the great potential of boric acid (BA)‐based fluorescent probes for glucose sensing, and the principle lies in the fluorescent quenching upon glucose binding to BA‐conjugated QDs.^[^ ^127^ ^]^ Li et al. prepared ratiometric fluorescent nanosensors by using BA‐conjugated QDs (BA‐QDs) and thioglycolic acid (TGA)‐coated QDs (TGA‐QDs) to realize the in vivo glucose sensing from single chloroplast to *Chara zeylanica* cells and *A. thaliana* leaf tissues (Figure 4d).^[^ ^128^ ^]^ In the presence of glucose, the Raspberry Pi camera system was used to image and record the visible fluorescence signal of BA‐QDs and TGA‐QDs. Results showed that the BA‐QDs fluorescence was quenched when the concentration of glucose exceeded 100 µM, while the fluorescence of TGA‐QDs remained invariant.

These genetically encoded nanosensors enable real‐time monitoring of transient, easily degradable, and low‐content metabolites, which is helpful for people to understand the dynamic changes of plant metabolic processes. But like the afore‐mentioned optical nanosensors, there are still some problems to be solved. First, the protein used should react with the specific substrate. And the gene must be able to be stably and correctly transcribed and translated in the organism without being degraded by the organism. This process is very time‐consuming and complex. Second, these sensors are difficult to apply to various plant species because of the greater differences between different species. Third, they are also difficult to measure multiple substances at the same time and their safety in plants needs to be further investigated. Finally, these sensors also need to carry out field trials.

## 3 Nanofertilizers for Plant Growth

**Figure 5:**
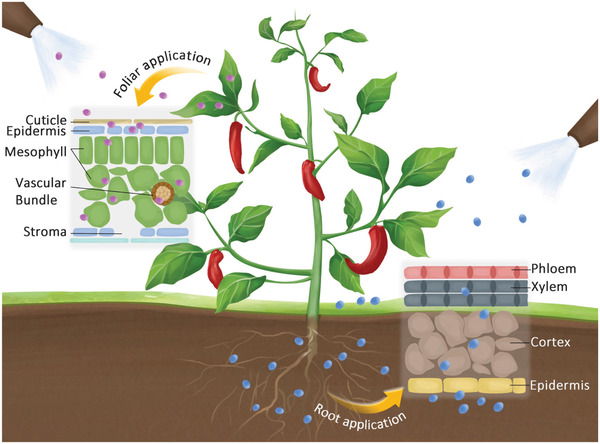
Nanofertilizers for plant growth.

Fertilizers are key to food production and still need to be used on a large scale because of the increasing food demand. Usually, plants absorb the applied nutrients inefficiently, leading to a sharp increase on farmers' costs. Nanotechnology has opened up many novel applications in the field of plant nutrition to promote plant growth, meeting the growing demand of food in the future. Its purpose is to increase the utilization efficiency of current fertilizers, either by increasing the efficiency of nutrients delivery to plants or by limiting the loss of nutrients in the environment.^[^ ^129^ ^]^ As shown in **Figure** **5**, nanofertilizers can be introduced into plants via foliar or root application. And they are engineered to be target oriented, thus improving nutrients utilization efficiency, decreasing the fixation of nutrients, and reducing nutrients loss.^[^ ^130^ ^]^ In general, depending on the role of the nanomaterials and the nutrients in use, we divide the nanofertilizers into four different categories: 1) Macronutrient nanofertilizers, 2) micronutrient nanofertilizers, 3) nanomaterials‐loaded nanofertilizers, and 4) other nanofertilizers. The first two categories of nanomaterials act as nutrients themselves for efficient uptake by plants. The third category of nanomaterials do not contain any essential plant nutrients, they often used as nanocarriers to slow release nutrients. And the last category of nanomaterials are not nutritionally required by plants, but they have been demonstrated to have positive impacts on plant growth and production.^[^ ^130^ ^]^

### 3.1 Macronutrient Nanofertilizers

Macronutrient nanofertilizers are composed of one or more macronutrient elements (e.g., (nitrogen) N, phosphorus (P), K, Ca, and sulfur (S)) to provide large amounts of nutrients required by plants. Global demand for macronutrient is estimated to reach 263 Mt by 2050.^[^ ^131^ ^]^ Especially for N fertilizer, it has increased per capita food production by about 40% in the past 50 years.^[^ ^132^ ^]^ However, these fertilizers (N, P, and K) are eventually transported into the surface and groundwater in large quantities, causing serious damages to the aquatic ecosystems.^[^ ^129^ ^]^ Therefore, it is necessary to develop high‐efficient and eco‐friendly macronutrient nanofertilizers realize sustainable food production on the basis of protecting the ecological environment.

N, P, and K are three major nutrients for plant growth and development. Among them, N is needed by the soil in the forms of solid or liquid including anhydrous ammonia, urea, ammonium, and nitrate.^[^ ^133^ ^]^ Different kinds of N, P, and K nanofertilizers, the fertilization method, and their growth enhancements have been listed in **Table** **1**, including N‐doped carbon dots, apatite nanoparticles (NPs), and monopotassium‐phosphate.^[^ ^23^, ^134^, ^135^ ^]^ The diameter of these nanofertilizers are very small (<20 nm), and they have good solubility in water. They are mixed with nutrient solution or water and applied to foliar or soil, which have greatly promoted the growth rate of plants. Moreover, composite nanofertilizers which can provide different nutrients (e.g., the monopotassium‐phosphate that can provide N, P, and K nutrients) alleviate the adverse effects of abiotic stress (e.g., salt stress) on plants.

**Table 1:** Currently reported macronutrient nanofertilizers

Nanomaterial	Comparison	Plant	Concentration	Fertilization method	Growth enhancements	Ref.
N‐CDs	Pure water and urea	Mung bean	0.2 mg L^−1^	Nutrient solution	The growth rate of mung bean improved by 200% (average length of shoots and roots).	[134]
Apatite NPs	Ca(H_2_PO_4_)_2_	Soybean	21.8 mg L^−1^	Soil	The growth rate and seed yield increased by 32.6% and 20.4%, and the biomass production was enhanced by 18.2% for the above‐ground and 41.2% for the below‐ground.	[135]
MKP	Nothing treated	Tomato	3 g L^−1^	Foliar and soil	The growth parameters of tomatoes under salt stress were improved.	[23]
CaO NPs	Bulk CaO and CaNO_3_	Groundnut	500 mg L^−1^	Foliar	The germination and growth rate of groundnut increased.	[24]
S NPs	Nothing treated	*Cucurbita pepo*	100–400 mg L^−1^	Soil	The number of leaves and branches, height per plant, stem diameter, and healthy plant increased.	[136]
S NPs	Nothing treated	Tomato	300 mg L^−1^	Soil	The root and shoot growth rate increased and the effect was concentration dependent.	[137]

Except for the above three major nutrients, Ca and S are also important macronutrients. Calcium oxide (CaO) NPs and S NPs have been utilized to mix with water and further applied to foliar and soil.^[^ ^24^, ^136^, ^137^ ^]^ However, these nanofertilizers have large and uneven diameters (20–80 nm), and they are used in relatively large quantities compared to the N, P, and K nanofurtilizers (Table 1). Nevertheless, it should be noted that, although these different nanofertilizers only consist of some nontoxic elements (i.e., C, O, N, K, P, Ca, and S), more thorough investigations are still needed to ensure their safety for large‐scale and long‐trem agricultural applications.

### 3.2 Micronutrient Nanofertilizers

Compared to macronutrients, micronutrients provide plants with relatively small amounts of essential nutrients (<10 mg kg^−1^ of soil).^[^ ^130^ ^]^ They are key elements to activate enzymes and the synthesize biomolecules involved in plant defense. Moreover, eating micronutrient‐deficient foods have adverse health effects on human health, leading to anemia, slower growth, and decreased cognitive.^[^ ^133^ ^]^ Therefore, as well as, macronutrient nanofertilizers, it is also necessary to apply micronutrient nanofertilizers to plants, including Zn, Cu, Iron (Fe), manganese (Mn), and molybdenum (Mo).

Zn, as an essential micronutrient, is required for plants, animals, and humans. Two studies used zinc oxide (ZnO) NPs as nanofertilizers, which were purchased from different companies.^[^ ^25^, ^138^ ^]^ Their particle sizes are about 50 and 70 nm, respectively. The dissolution of these ZnO NPs in water is relatively slow. But the diameter of these ZnO NPs or their aggregates still smaller than the stomatal pore size, exhibiting the ability to penetrate and move inside plant tissues. After the NPs were attached to leaf surfaces, Zn^2+^ might be continuously released, providing a long‐term source of Zn that can be absorbed by plants through stomatal openings. Therefore, the application of ZnO NPs improved the growth and yield of different plants.

Fe is an important nutrient involved in chlorophyll biosynthesis and electron transfer system, and Fe deficiency can affect the normal physiological function of plants and reduce the nutritional quality.^[^ ^139^ ^]^ Palmqvist and colleagues synthesized the maghemite (*γ*‐Fe_2_O_3_) NPs to serve as nanofertilizers.^[^ ^140^ ^]^ Results showed that the drought resistance of the plants was greatly enhanced. Furthermore, the H_2_O_2_ was reduced, the growth rate of leaves and the chlorophyll content were increased. Although, the dissolution of *γ*‐Fe_2_O_3_ NPs is slow and the NPs form aggregates, with a hydrodynamic size of up to 500 nm. This is also conducive to the long‐term sustained absorption of iron ions (Fe^3+^) by plants. In another study, Liu and coworkers utilized nanoscale zero‐valent iron (nZVI) to realize soil remediation simultaneously with increasing rice production.^[^ ^141^ ^]^ They found that nZVI with bigger size (100 nm) and lower coercivity (35.17 Oe) could greatly improve grain yield and the removal rate of pollutants in the soil. The other nZVI with smaller size and higher coercivity exhibited greater homoaggregation in soil, which have negative impacts on their performance.

Cu is another essential micronutrient for plants and microbial growth. Different Cu‐based NPs, including CuO, copper sulphide (CuS), and copper hydroxide (Cu(OH)_2_)) have been applied to root and foliar of different plants (**Table** **2**).^[^ ^26^, ^142^ ^]^ Among them the spindle‐shaped Cu(OH)_2_) NPs exhibit higher dispersibility than spherical CuO and CuS NPs, thereby providing more uptake of Cu to plants. However, the CuO and CuS NPs with lower dispersibility are more persistent on the roots and continued to transport Cu to plant leaves during the application. Mn is also an important micronutrient for plants and is mainly involved in photosynthesis. The hydrodynamic radius of stable square shaped Mn NPs is about 100 nm, which can be stably dispersed in water, and the size of the dispersed particles remains within the nanoscale range. This Mn NPs‐based nanofertilizers are nontoxic even at a higher concentration, and their smaller size help plants to uptake these particles more readily.^[^ ^143^ ^]^

**Table 2:** Currently reported micronutrient nanofertilizers

Nanomaterial	Comparison with	Plant	Concentration	Fertilization method	Growth enhancements	Ref.
ZnO NPs	Nothing treated	Rice (PR‐121)	5 g L^−1^	Foliar	The growth, yield, yield‐attributing characters, microbial counts, and the dehydrogenase enzyme activity improved.	[25]
ZnO NPs	ZnSO_4_ and untreated group	Coffee (*Coffea arabica* L.)	10 mg L^−1^	Foliar	The fresh weight (roots: 37%, leaves: 95%), dry weight (roots: 28%, leaves: 85%), and the net photosynthetic rate (55%) increased.	[138]
*γ*‐Fe_2_O_3_ NPs	FeCl_3_	*Brassica napus*	2 mg mL^−1^	Soil	The H_2_O_2_ content reduced to 83 µM g^−1^, the malondialdehyde formation reduced to 26 mm g^−1^, growth rate of leaves enhanced to 50%, and chlorophyll content increased to 52.	[140]
nZVI	mZVI and Fe^2+^	Rice	100 mg kg^−1^	Soil	The grain yield increased (47.1–55.0%), the grain PCP content decreased (83.6–86.2%), and the soil PCP removal rate increased (49.9–89.0%) after the addition of three different nZVI.	[141]
Cu‐based NPs (CuO, CuS, and Cu(OH)_2_)	Nothing treated	Wheat (*Triticum aestivum*)	1 mg mL^−1^	Root	High‐solubility Cu(OH)_2_ NPs provided more uptake of Cu, while low‐solubility materials (CuO and CuS) were more persistent on the roots and continued to transport Cu to plant leaves during the 48 h depuration period.	[142]
CuO NPs	Nothing treated	Maize (*Zea mays* L.)	8 mg L^−1^	Foliar	The plant growth grate improved by 51%.	[26]
Mn NPs	MnSO_4_	Mung bean (*Vigna radiata*)	0.05 mg L^−1^	Foliar	The root and shoot length increased by 52% and 38%, respectively, and the fresh and dry weight enhanced.	[143]
Mo NPs	Water treated	Chickpea (*Cicer arietinum* L.)	8 mg L^−1^	Seed	The microbial activity and seed growth improved.	[296]

### 3.3 Nanomaterials‐Loaded Nanofertilizers

Nanomaterials‐loaded nanofertilizers can promote plant growth to some degree, although these nanomaterials cannot be well absorbed by plants. They are used as nanocarriers for sustained delivery of nutrients, thereby increasing plant uptake efficiency of the nutrients and reducing the adverse effects of traditional fertilizers. Typical examples of this type are chitosan NPs and nano‐zeolite.

Relative to bulk chitosan, chitosan NPs combine the characteristics of chitosan and the properties of NPs like surface and interface effect, excellent physicochemical properties, highly soluble in aqueous media, and environmentally friendly, as well as, bioactive.^[^ ^130^, ^144^, ^145^, ^146^, ^147^ ^]^ Therefore, chitosan NPs are often used as nanocarriers for loading NPK to realize the slow release of NPK fertilizers. According to literature reports, chitosan molecules in the solution are in the form of cationic polyelectrolytes, which are easy to form specific nanostructures through the electrostatic interaction with methacrylic acid (MAA).^[^ ^148^ ^]^ Therefore, most of the chitosan NPs used as nanocarriers are formed by the polymerization of MAA in the chitosan solution (i.e., CS‐PMMA NPs).^[^ ^148^, ^149^ ^]^ For example, Abdelaziz et al. investigated the effects of CS‐PMMA NPs loaded with NPK fertilizers on the wheat grains through foliar application.^[^ ^145^ ^]^ They found the total saccharide, K, and P contents in the wheat grains were significantly increased when compared with common fertilizers. Khailfa and colleagues used the same nanomaterial to load NPK fertilizers and evaluate their effect on garden pea plants (*Pisum sativum* var. Master B).^[^ ^150^ ^]^ They found that compared with other groups, the prepared nanofertilizer could induce mitosis, and the expression of some major proteins in plants was also upregulated. The average diameter of the CS‐PMMA NPs are about 23 nm. After loading NPK, the dispersion of NPs is homogenous without any agglomeration, and it has good stability at different pH gradients. Although the slow release of NPK fertilizers with the help of chitosan NPs has been achieved, the potential impact of such systems on agriculture remains to be further explored, as their accumulation in the field may have negative effects on plants and the environment.

In general, silicon (Si) and aluminum (Al) are arranged in the 3D framework of silicon oxygen tetrahedron and aluminum oxygen tetrahedron of zeolites, respectively, forming channels and voids at nanometer scale (0.3–10 nm diameter).^[^ ^129^ ^]^ Therefore, zeolite is a kind of material with nanostructures. Studies have shown that nano‐zeolite, as a nanocarrier of common fertilizers, can increase plant yields and reduce the use of fertilizers.^[^ ^151^ ^]^ The nano‐zeolite is typical cubic to round in shape with the size of 90–100 nm. After loading with Fe_2_O_3_ and ZnSO_4_ NPs, the nano‐zeolite help to increase plant yield by slow and long‐term releasing of Fe^3+^ and Zn^2+^, greatly increasing the use efficiency of Fe and Zn.^[^ ^152^, ^153^ ^]^

### 3.4 Other Nanofertilizers

In addition to the three types of nanofertilizers mentioned above, some nanomaterials that are not classified as plant nutrients also have positive impacts on plants. Although these nanomaterials themselves are not nutrient elements that required by plants, they still can promote plant growth and development. This group of nanomaterials include titanium dioxide NPs (TiO_2_ NPs), CNTs, and ceric dioxide NPs (CeO_2_ NPs).

Ti is generally not considered as an essential plant nutrient. However, some studies indicated that TiO_2_ NPs could improve the growth of plants by enhancing photosynthesis.^[^ ^154^ ^]^ One study synthesized TiO_2_ NPs (12–15 nm) evaluated its effect on mung bean growth.^[^ ^155^ ^]^ After the foliar application, significantly improvements were observed in shoot and root length, chlorophyll content, and total soluble leaf protein. The TiO2 NPs may adsorb to plant leaf surfaces and taken up through stomatal openings. After being absorbed by plants, the TiO_2_ NPs may increase the activity of phytase and phosphatase enzyme, thereby helping in native phosphorous nutrient mobilization in rhizosphere and enhancing plant metabolic activities.

Some studies have shown that CNTs have the ability to penetrate the cell walls and membranes of plants.^[^ ^156^ ^]^ Usually at low doses, CNTs can stimulate seed germination and plant growth. Joshi et al. assessed the effects of multi‐walled carbon nanotube (MWCNT, diameter of 35 nm and lengths of 200–300 nm) on the growth and yield of oat.^[^ ^157^ ^]^ They found that through the seed‐priming method, these MWCNT traversed the cells and enhanced the growth rate of xylem cells, chlorophyll content, and photosynthetic activity. Moreover, the MWCNT has no toxic effects on the DNA of the plants, which makes a lot of sense for its application.

Besides TiO_2_ NPs and CNTs, there are also many studies about the influence of CeO_2_ NPs on plants. The CeO_2_ NPs with a primary size of about 8 nm and will aggregate to about 240 nm in water. After applying to the roots or leaves of plants, they help to increase growth rate and yield.^[^ ^158^ ^]^ Moreover, they also have better performances in inhibiting Fusarium wilt and increasing chlorophyll content in plants.^[^ ^158^ ^]^ However, more investigations need to be conducted to examine the long‐trem effect of Ce on plant quality.

For all nanomaterials that used to enhance plant growth, the mechanism of their interaction with plants needs to be thoroughly investigated, otherwise their long‐term accumulation in plants could pose threats to animal and human health. Future researches also need to further explore the influence of size, shape, charge, and solubility of nanomaterials on their performances and plants.

## 4 Nanopesticides for Plant Protection

**Figure 6:**
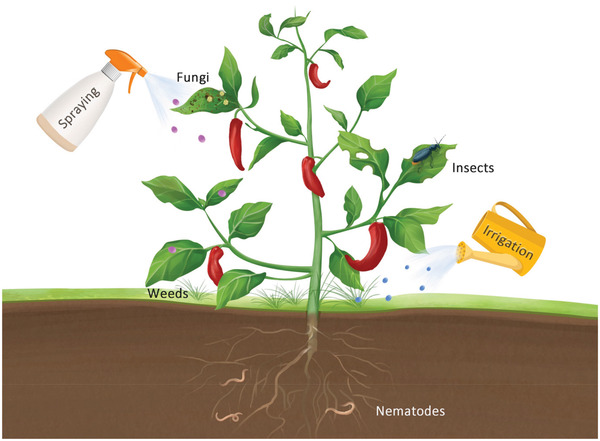
Nanopesticides for plant protection.

Effective pest management is necessary for agricultural production. However, it is estimated that about 90% of the applied chemical pesticides are lost during or after application due to volatilization, degradation, and photolysis, which have serious effects on food chain and human health.^[^ ^159^, ^160^ ^]^ In addition, the widespread use of pesticides has led to increased pesticide‐resistance in weeds, insects, and pathogens. Therefore, the use of pesticides should be controlled in an efficient and eco‐friendly way, and can be effectively delivered to specific sites.^[^ ^161^ ^]^ Since nanotechnology has been proven in large amounts of experiments to improve plant growth and increase nutrient utilization efficiency, their potential in protecting plants from pests, pathogens, weeds, etc., is also gaining increasingly interests.^[^ ^162^, ^163^ ^]^ Nanopesticides, which can be defined as any pesticide formulations that contain nanomaterials with biocidal properties, have been widely studied (**Figure** **6**). In general, nanomaterials itself can be directly used as pesticides, and they also can be used to protect the pesticides and enhance their delivery to the site of action. Therefore, in this section, we divide the nanopesticides into two categories: 1) Nanomaterials directly used as nanopesticideds and 2) nanomaterials used as nanocarriers for pesticides.

### 4.1 Nanomaterials Directly Used as Nanopesticides

The broad spectrum of antifungal/antibacterial properties of nanomaterials allow them to be directly applied to plant seeds, leaves, and roots to against pest and pathogens. They also solve the problem of the increases in microbial resistance after the application of traditional chemical pesticides for a period of time. Among them, metal‐based NPs such as Ag, Cu, Mg, Zn, and Ti, have been extensively investigated. Besides, S and Si NPs also exhibit antimicrobial activities with low toxicity to animals and humans. In this section, a brief overview about the NPs with biocidal properties is given (also **Table** **3**).

**Table 3:** Currently reported nanopesticides

Nanomaterial	Comparison with	Plant	Concentration	Application type	Targets	Growth enhancements	Ref.
Ag NPs	Nothing treated	Bermudagrass	150 mg L^−1^	Soil	Nematode	The number of nematodes reduced by 82% and 92% after 4‐ and 2‐day, respectively.	[164]
Ag@dsDNA@GO	Nothing treated and treated with copper (Kocide 3000) and mancozeb (Penncozeb 75DF)	Tomato	100 mg L^−1^	Soil	*Xanthomonas perforans*	The severity of bacterial spot disease reduced with no phytotoxicity.	[167]
GO‐Ag NPs nanocomposite	Pure Ag NPs and GO suspension	Wheat	9.37 µg mL^−1^	Foliar	*Fusarium graminearum*	The inhibition efficiency of *Fusarium graminearum* was threefold and sevenfold higher than pure Ag NPs (12.45 µg mL^−1^) and GO suspension (250 µg mL^−1^).	[166]
Ag NPs	Nothing treated	Rice	100 mg L^−1^	Foliar	Xoo	The bacterial leaf blight of rice caused by Xoo reduced and the plant biomass increased.	[165]
Cu_3_(PO_4_)_2_·3H_2_O nanosheets	Commercial CuO NPs	Watermelon	10 mg mL^−1^	Foliar	*F. oxysporum* f. sp. *niveum*	The Cu_3_(PO_4_)_2_·3H_2_O at a lower concentration significantly suppressed fungal disease compared with control groups.	.^[^ 173]
rGO‐CuO	Chemical fungicide (Kocide 2000) and water	Tomato and pepper	1 mg L^−1^	Seed	*F. oxysporum*	The rGO‐CuO NPs treated plants exhibited beneficial effects on flowering, plant height, dry weight, and the accumulation of photosynthetic pigments.	[172]
CuO NPs	Nothing treated	Bt‐transgenic cotton	10 mg L^−1^	Nutrient solution	Bollworm	The Bt toxin protein in the leaves and roots enhanced and the pest resistance of transgenic insecticide crops improved.	[169]
MgO NPs	Distilled water	Tomato	0.7% suspension	Root	*Ralstonia solanacearum*	The treated tomatoes showed great inhibition of bacterial wilt.	[174]
Mg(OH)_2_ NPs	Commercial pesticide (Kocide 3000) and distilled water	Tomato	1000 µg mL^−1^	Foliar	*X. alfalfae*, *P. syringae*, and *E. coli*	Mg(OH)_2_ NPs exhibited comparable bacterial killing efficacy and reduced phytotoxicity on tomato leaves and seeds.	[175]
ZnO NPs	Acibenzolar‐S‐methyl, streptomycin sulfate, and nothing treated	Sweet orang	4.14 mg per plant	Foliar	*Xanthomonas citri* subsp. citri	The citrus canker lesion development (38% in trials 1 and 42% in trials 2) of sweet orange that inoculated with *Xanthomonas citri* subsp. Citri reduced.	[176]
TiO_2_ NPs, TiO_2_/Zn NPs, and TiO_2_/Ag NPs	Nothing treated and Cu/mancozeb‐treated	Tomato	500–800 mg L^−1^	Foliar	*Xanthomonas perforan*	The bacterial spot disease severity of tomatoes reduced.	[177]
S NPs	Nothing treated	Tomato	0.704 mg mL^−1^	Foliar	*Fusarium solani* and *Venturia inaequalis*	The smaller S NPs (≈35 nm) obtained higher growth inhibition toward *Fusarium solani* and *Venturia inaequalis*.	[181]
S NPs	Cu NPs and chitosan NPs	Ginger	100 µg mL^−1^	Rhizome	*F. oxysporum*	The S NPs could be used for effective management of *F. oxysporum* that causes soft‐rot of gingers.	[182]

Silver NPs (Ag NPs) is a broad‐spectrum antimicrobial agent that can affect plant pathogenic bacteria and fungi. Chemical reduction is the most commonly used method for preparing Ag NPs, which is obtained by reducing silver nitrate (AgNO_3_) with different reducing agents. Cromwell et al. prepared Ag NPs via the redox reaction between silver nitrate (AgNO_3_) and sodium borohydride, which could be used as a nematicide.^[^ ^164^ ^]^ Besides, the green synthesis method of Ag NPs has been increasingly advocated recently, and most of them use plant or bacterial extracts to reduce AgNO_3_. One study has confirmed the antibacterial activity of green Ag NPs against *Xanthomonas oryzae* pv., *oryzae* (Xoo), which is the most destructive pathogen of rice.^[^ ^165^ ^]^ The two studies demonstrate that Ag NPs can increase the activity of antioxidant enzymes of plants to activate the antioxidant system, thereby reducing the ROS caused by biotic or abiotic stresses. However, the aggregation and settling of pure Ag NPs may occur due to its strong adhesion forces between particles with high surface energy, leading to the decrease of antimicrobial activity. Thus, some measures need to be taken to stabilize Ag NPs or control the slow‐release of Ag ions. GO sheet with special surface properties, large surface area, and high water solubility, which can solve the above problems well by acting as dispersion and surfactant‐like agents.^[^ ^166^ ^]^ This GO‐Ag NPs nanocomposite was prepared through interfacial electrostatic self‐assembly, where the Ag NPs were homogeneously attached to the surface of GO sheets and the solution of GO‐Ag NPs nanocomposite was still well distributed at a higher concentration. Due to the synergistic effect between GO and Ag NPs, they exhibited good antibacterial activity, where the inhibition efficiency against *Fusarium graminearum* was threefold higher than pure Ag NPs. In another, dsDNA was used as a stabilizer for further enhancing the synergistic effect and the water solubility of the nanocomposite.^[^ ^167^ ^]^ Here, the dsDNA is beneficial to control the size and distribution of Ag NPs on the surface of GO, and avoid the aggregation of NPs at the same time. This Ag@dsDNA@GO‐based nanopesticides exhibited better inhibition efficiency against *Xanthomonas perforans* than pure Ag NPs and GO‐Ag NPs nanocomposite. Although these Ag NPs‐based nanopesticides exhibit no phytotoxicity, their long‐term efficacy in the field still needs to be further investigated. Whether their continuous accumulation in the field will threaten human health through the enrichment of the food chain is still unclear.

Besides Ag NPs, the antibacterial and antifungal properties of Cu ions are also outstanding.^[^ ^168^, ^169^ ^]^ Compared with bulk Cu, CuO NPs exhibit better catalytic activity and inhibition of microorganism.^[^ ^170^ ^]^ Van et al. studied the effects of CuO NPs (30 ± 10 nm) on *Bacillus thuringiensis*‐transgenic (Bt‐transgenic) cotton.^[^ ^169^ ^]^ They founs that CuO NPs could improve the expression of the Bt toxin protein both in leaves and roots even at low concentrations, thereby increasing the resistance of crops to pests. However, like pure Ag NPs, the pure CuO NPs are prone to agglomerate during use, which leads to poor antifungal activity.^[^ ^171^ ^]^ Therefore, in another study, the researchers prepared rGO‐CuO nanocomposite through chemical precipitation method, and studied its antifungal activity against wild strains of *Fusarium oxysporum*.^[^ ^172^ ^]^ With the help of rGO, the CuO NPs become more stable and more soluble in water. And the antifungal activity of the rGO‐CuO nanocomposite with smaller size of CuO NPs (5 nm) was the best. That is because the smaller NPs have a larger surface area, thereby promoting more interactions with the fungal cells as well as accelerating the release of Cu ions. Besides CuO NPs, cupric phosphate anhydrous (Cu_3_(PO_4_)_2_·3H_2_O) nanosheets also exhibit antifungal activity against *F. oxysporum*.^[^ ^173^ ^]^ Moreover, compared with pure CuO NPs, the Cu_3_(PO_4_)_2_·3H_2_O nanosheets with the properties of smaller particle size, unique particle structure (nanosheets), and faster initial ions release rate, showing broad application prospects in agriculture. Therefore, the difference in particle composition and size, as well as the release rate of Cu ions, will affect the efficacy of Cu‐based nanopesticides.

Ag or Cu‐based NPs have been extensively applied to control a broad spectrum of crop diseases, but their prolonged use can lead to the development of resistance in certain plant pathogens. Thus, other metal‐based nanomaterials, such as, Mg, Zn, Ti, etc., have been extensively studied. Among them, Mg‐based NPs with the advantages of nontoxic and easy‐prepared show great potential in acting as bactericidal materials. By treating the tomato roots with MgO NPs suspension, the treated plants have a strong inhibition of bacterial wilt.^[^ ^174^ ^]^ Moreover, this MgO NPs‐based nanopesticides are harmless to environment and low‐cost, thereby having a broader application prospect. Besides MgO, magnesium hydroxide NPs (Mg(OH)_2_ NPs) also exhibited comparable bacterial killing efficacy and reduced phytotoxicity on tomato leaves and seeds.^[^ ^175^ ^]^ This study demonstrates that the Mg(OH)_2_ NPs have greater potential in crop protection that can replace Cu‐based fungicides, as they show comparable bactericidal efficacy and lower phytotoxicity. For Zn and Ti‐based NPs, they also play roles in pest management. ZnO NPs‐based nanopesticides could significantly reduce the citrus canker of sweet orange that inoculated with *Xanthomonas citri* subsp. Citri.^[^ ^176^ ^]^ And TiO_2_ NPs with intrinsic photochemical activity exhibited high photocatalytic activity against *X. perforans* within 10 min.^[^ ^177^ ^]^ Moreover, the TiO_2_ doped with Zn (TiO_2_/Zn NPs) performed well in the greenhouse studies, significantly reducing the bacterial spot disease severity of tomatoes.

From the above studies, we found that in addition to being used as pesticides, Cu, Mg, and Zn are plant nutrients themselves, which play a role in promoting growth, activating enzyme activity, participating in photosynthesis, and other physiological processes. This can serve as a reference for future research work in this field. Besides these metal‐based nanomaterials, S NPs are also a plant macronutrient, and they also have great inhibitory effects against several microorganisms.^[^ ^178^, ^179^, ^180^ ^]^ Novel green synthetic routes for the preparation of S NPs were developed and proved their antifungal effects on different phytopathogens, for example, *Fusarium solani*, *Venturia inaequalis*, and *F. oxysporum* have been studied.^[^ ^181^, ^182^ ^]^ Results showed that the smaller S NPs had a stronger inhibitory effect on fungal growth than larger NPs. Consider the excellent performance of these NPs in pest management. And some of them are plant nutrients with green nature, which can reduce the toxic effects of chemical pesticides in farmland. However, it is still necessary to evaluate the toxicity of these NPs in human beings and environment to generate more information to clearly understand the toxic effects, if any. For example, although Ag has been widely used due to its broad‐spectrum bactericidal property, Ag can also cause argyria in mammalian cells.^[^ ^183^ ^]^

### 4.2 Nanomaterials Used as Nanocarriers for Pesticides

As for pest management, it is also important to realize the controlled delivery of pesticides with enhanced activity at low drug concentrations.^[^ ^184^ ^]^ Nanocarrier, which is composed of a shell or membrane structure to encapsulate pesticides inside, is an effective tool for protecting pesticides from harsh environment. Nanocarrier can increase the chemical stability, dispersion, and wettability of pesticides, thus significantly lowering the risk of organic solvent runoff.^[^ ^21^ ^]^ And the smart delivery systems based on nanocarriers can release pesticides to the target site and improve the penetration of the pesticides into the targeted tissues. In this section, several nanomaterials for the encapsulation of pesticides are discussed in details, including polymeric nanomaterials, solid lipid NPs (SLNPs), Si NPs, and metal organic frameworks (MOFs).

Polymeric nanomaterials, with the properties of biocompatibility and minimal side effects on non‐target parts, are the most widely utilized nanocarriers for pesticides.^[^ ^185^ ^]^ They usually include synthetic (polyacrylate, polyethyleneglycol (PEG), polycaprolactone (PCL), polylactic acid (PLA), natural (chitosan, sodium alginate, collagen, etc.) polymers, etc. For hydrophobic PLA microspheres can effectively prevent water from penetrating the polymer, which is the key to allowing pesticides to diffuse from the microspheres. At the same time, the addition of PCL increases the hydrophilicity of PLA, thereby improving the diffusion ability of pesticides.^[^ ^186^ ^]^ Therefore, such amphiphilic polymers can be used as a container to encapsulate pesticides in the polymeric matrix surrounded by a hydrophilic shell, which maximizes the exposed surface area of pesticides and greatly reduces the actual amount of pesticides used. Moreover, the encapsulated pesticides can also be lyophilized for storage. For water‐soluble PEG, it can be used as a filler to assist other polymers to encapsulate pesticides, promoting the rapid release of pesticides and preventing the aggregation of other polymers through van der Waals interactions and hydrophobic interactions.^[^ ^187^, ^188^ ^]^ Besides these synthetic polymers, natural polymers like chitosan also play an important role in promoting the accumulation and controlled release of pesticides to target sites.^[^ ^189^, ^190^ ^]^ The encapsulation of fungicide in nontoxic chitosan helps reduce the phytotoxicity of fungicide. This is due to the biocompatibility, biodegradability, and non‐toxicity of natural biopolymer. Moreover, for some pesticides with poor water solubility, photostability, and durability like avermectin (AVM), they can be well encapsulated in chitosan NPs through the electrostatic interaction, thereby prolonging the leaching time of AVM and enhancing its utilization efficacy.

SLNPs are composed of lipids that are solid phase at room temperature, which are similar to emulsions. They have been reported as promising nanocarriers of various lipophilic components with the advantages of reduced chemical degradation of pesticides and feasible large‐scale production.^[^ ^21^, ^191^, ^192^, ^193^ ^]^ The SLNPs also have been demonstrated to have the ability to overcome the photodegradation of pesticides without any application of UV absorbers. For pesticides that are extremely prone to photodegradation in the natural environment, such as, deltamethrin, they are effectively protected when they are loaded in the SLNPs.^[^ ^194^ ^]^ Besides showing good protection for pesticides against photodegradation, SLNPs can also offer high colloidal stability to encapsulate two kinds of herbicide (atrazine and simazine).^[^ ^195^ ^]^ The encapsulation efficiency of both herbicides is very high, and they are well dispersed throughout the nanoparticle matrix and interact with the lipid matrix. SLNPs encapsulated with two different herbicides not only improve the efficiency of them, but also reduce the toxicity of the active agents to nontarget organisms and the environment.

For Si NPs, they can be easily synthesized with different shapes, sizes, and structures, making them useful nanocarriers to protect pesticides against degradation by UV light.^[^ ^196^, ^197^ ^]^ Most of the Si NPs show a spherical shape with pore‐like holes, where the mesoporous Si NPs (MSNs) are typical examples. MSNs have the characteristics of controllable mesoporous structure, large pore volume, and easy surface modification.^[^ ^198^ ^]^ The inner core of Si NPs can be used to load pesticides and the outer shell structure can protect them against UV light, thereby regulating the release of pesticides and avoiding the premature release of pesticides before reaching the target site. One study has demonstrated that the MSNs could effectively prevent pesticides degradation by holding them in their mesoporous structure and improved the foliar uptake, deposition, translocation, and distribution of pesticides in plants.^[^ ^199^ ^]^ As we all know that some properties of functionalized nanomaterials can be improved. But for MSNs, functional modification for MSNs before loading pesticides might cause the blockage of the pore channels and the reduce of specific surface area, thereby reducing the loading content.^[^ ^200^, ^201^ ^]^ Satisfactory loading content can be maintained if the pesticide encapsulation and surface modification are carried out synchronously. This synchronous preparation method also provides ideas for future work.^191^

Recently, MOFs, a kind of organic‐inorganic hybrid material, have gained significant attention to act as nanocarriers due the advantages of large surface area, regular porosity, and stable structure.^[^ ^202^, ^203^ ^]^ They are also easy to be functionalized and have the properties of water solubility and biodegradability. Yang et al. synthesized porous and chiral MOFs based on Ca^2+^ and lactate to encapsulate fumigant.^[^ ^204^ ^]^ Results showed that the release rate of fumigant‐loaded MOFs was 100 times lower than liquid fumigant. Moreover, the MOFs are biodegradable, there will be no accumulation issues in the environment, and only Ca will be left in the soil as nutrient. However, the MOFs that act as nanocarriers for pesticides usually tend to release a small amount of pesticides at the early stage, where such a concentration of pesticide is far from enough to effectively kill pests.^[^ ^205^ ^]^ To solve this problem, Huang et al. synthesized a novel double‐layer nanocarrier to load thiamethoxam, which was formed through the chemical crosslinking between the sulfonic acid groups on sodium lignosulfonate (SL) and protonated UIO‐66‐NH_2_ (UIO‐66‐NH_2_/SL).^[^ ^206^ ^]^ This method not only overcomes the shortcomings of too slow early release of MOFs‐based nanocarriers, but also solves the problem that some natural polymer carriers in the soil cannot be burst release. In another study, considering that some MOFs with heavy metals (e.g., Zr, and Cr) may pose threats to the environment, Liang et al. used zeolitic imidazolate framework‐8 (ZIF‐8), which is composed of 2‐methylimidazole (2‐mim) and Zn^2+^ with relatively low cytotoxicity, to encapsulate pesticides through a one‐pot method.^[^ ^207^ ^]^ Since ZIF‐8 is easy to decompose under acidic conditions, it realizes the on‐demand release of pesticides in acidic microenvironment, thereby constructing a pH‐responsive delivery systems in agriculture.

From the aforementioned studies, we can conclude that the following objects need to be met for the effective application of nanomaterials as nanocarriers for pesticides in agriculture: i) Increase the solubility of active agents in pesticides, ii) realize controlled slow release of pesticides to eliminate pests completely, iii) protect pesticides from premature degradation caused by certain factors in the environment (e.g., UV radiation), iv) reduce the toxicity of the active agents to nontarget organisms and the environment, and v) the nanocarriers themselves are biocompatible and stable. Compared with traditional systems, the use of nanocarrier systems has unique advantages. They increase the biological activity of active agents, reduce the amount of necessary pesticides, and alleviate environmental pollution and human health threats. However, a large number of long‐term field trials are still needed to clarify the uptake and translocation of these nanocarriers in plants, thereby realizing sustainable agricultural production. Moreover, for large‐scale applications, simple and green preparation methods are required.

## 5 Nanomaterials‐Mediated Plant Gene Transformation

The development of plants that are resistant to pests intrinsically and have high yields without excessive use of pesticides and fertilizers is essential. Thus, genetic enhancement of plants has been proposed, which is a powerful supplement to the traditional breeding methods. Conventional methods for biomolecules delivery in plants include *Agrobacterium*‐mediated transformation and gun particle bombardment, which usually have the disadvantages of low efficiency, limited biomolecules and plant types, complex process, random DNA integration, and tissue damage.^[^ ^208^ ^]^ And there has yet to be a plant transformation method that realizes the efficient gene delivery without the need of transgene integration.^[^ ^38^ ^]^ This is because genetically modified organisms (GMOs) have been controversial around the world, and views on genetically modified foods in different countries are extremely polarized. Moreover, unlike animal cells, plant cells have a multilayered and rigid cell wall that composed of cellulose microfibrils. Exogenous biomolecules are difficult to traverse the rigid cell walls of plants without the help of external forces. Therefore, for plant genetic engineering, the successful delivery of biomolecules across the plant barriers without the aid of external force and without causing tissue damage is a great challenge.^[^ ^33^, ^209^ ^]^ In addition, we also need to find a method that can circumvent strict GMO regulations while achieving efficient genome modification without transgene integration.

In recent years, nanocarriers have been widely studied in animal cells, and their potential for plant systems is a more recent undertaking.^[^ ^34^, ^210^, ^211^, ^212^ ^]^ The size exclusion limit (SEL) of plant cell wall between 5 and 20 nm is the main obstacle to the delivery of exogenous biomolecules.^[^ ^213^ ^]^ The SEL of plant cell membranes is larger (about 300 to 500 nm), which are additional obstacles.^[^ ^214^ ^]^ And the charge and shape of nanocarriers greatly cell membrane translocation, for example, cationic NPs are more likely to bind to negatively charged cell membranes, thereby the internalization of cationic NPs is more effective than anionic NPs.^[^ ^215^ ^]^ Moreover, it is also necessary to consider plant physiology, because different plant species may have totally different physiological characteristics.^[^ ^216^ ^]^ Recently, nanomaterials have attracted a lot of attention in plant science because of their unique characteristics as compared to their bulk counterparts, and many related researches have been mentioned above. For plant genetic engineering, nanomaterials with the properties of tunable size (to accommodate the pore diameter of plant cell wall, adjustable between 5 and 20 nm) and physicochemical features allow them to penetrate plant barriers and conjugate with different biomolecules, thereby introducing desired traits into plant species.^[^ ^217^ ^]^ Various studies have demonstrated that CNTs,^[^ ^38^, ^43^, ^98^ ^]^ MSNs,^[^ ^218^, ^219^, ^220^ ^]^ QDs,^[^ ^221^ ^]^ magnetic NPs (MNPs),^[^ ^222^, ^223^ ^]^ etc., can enter the plant through root tissues (e.g., root tips, rhizodermis, etc.) or aboveground organs/tissues (e.g., stomata, cuticles, stigmas, hydathodes, etc.).^[^ ^216^ ^]^ After penetrating into plants, nanomaterials can move in tissues through the apoplast pathway (via extracellular spaces, cell walls, and xylem vessels) or the symplast pathway (via sieve plates and plasmodesmata).^[^ ^215^, ^224^, ^225^ ^]^ Along these different pathways, nanocarriers can protect the carried biomolecules from cellular metabolism and degradation.^[^ ^38^ ^]^

Currently, the potential role of various nanomaterials in plant genetic engineering has been studied. And as an emerging field, the nanomaterials‐based delivery platform for exogenous biomolecules is expected to play an important role in plant genetic engineering and genome editing. Here, we summarize different types of nanomaterials that have been utilized to deliver biomolecules in plants and the progresses made so far (also **Table** **4**). The commonly used nanomaterials to deliver biomolecules discussed below include CNTs, MSNs, metal‐based NPs, MNPs, QDs, and other nanomaterials that used as biomolecules delivery platform.

**Table 4:** Currently reported nanomaterials‐mediated gene delivery platforms in plants

Nanomaterial type	Plant species, cell or tissue type	Delivery type	Delivery method	Outcomes	Ref.
SWCNTs/MWCNTs	*N. tabacum* protoplasts and leaf explants	YFP plasmid	Co‐culture	The efficiency of callus formation and regeneration of shoots from the callus with *N. tabacum* leaf explants reached 100% on the third week of cultivation on the medium.	[228]
SWCNTs	Leaves of tobacco	Cy3‐siRNA	Needleless injection	SWCNTs efficiently delivered siRNA and silence endogenous genes (GFP) in intact plant cells and protected siRNA from nuclease degradation.	[41]
SWCNTs/MWCNTs	*Nicotiana benthamiana* (tobacco), *Eruca sativa* (arugula), *T. aestivum* (wheat), and *Gossypium hirsutum* (cotton) leaves	GFP‐ and Cy3‐pDNA	Needleless injection	The CNTs‐based platform delivered pDNA in species‐independent way without mechanical aid and transgene integration.	[38]
CS‐SWCNTs	Chloroplasts of mature *E. sativa* (arugula), *Nasturtium officinale*, *Nicotiana tabacum* (tobacco), *Spinacia oleracea* (spinach) plants, and isolated *A. thaliana* mesophyll protoplasts	pDNA	Needleless injection	The CS‐SWCNTs penetrated the protoplast and chloroplast membranes and localized within the chloroplasts, where the YFP expression was observed.	[39]
IM‐SWCNTs	Oil palm pollen grains	pDNA	Passively delivery	The IM‐SWCNTs with high biocompatibility successfully delivered pDNA into oil palm pollen grains.	[232]
MSNs	Roots of *A. thaliana*	pDNA	Co‐culture	The TMAPS/F‐MSNs passed through the cell wall barrier and moved to the deep tissuesand several organelles.	[234]
MSNs	Leaves and roots of tomatos	pDNA	Spray and needleless injection	The MSNs‐based transient expression system successfully transferred the exogenous genes into plants and further performed the transcription and translation steps in the target plant tissues.	[220]
mGNPs	*B. napus* protoplasts and cell suspension	*β*‐Glucuronidase (GUS)	Magnetic field	The blue colour appeared in the canola protoplasts and walled canola cells, indicating successful GUS gene expression.	[222]
DNs	Leaves of *N. benthamiana*	siRNA	Needleless injection	Leaves infiltrated with siRNA‐DNs exhibited the largest decrease in GFP fluorescence intensity when compared with the control groups.	[213]
LDH clay nanosheets	Leaves of *N. tabacum* cv. Xanthi	dsRNA	Leaf spraying	The BioClay provided a longer protection window on *N. tabacum* cv. Xanthi leaves that challenged with PMMoV than naked dsRNA.	[252]
LDH clay nanosheets	Leaves of *N. benthamiana* and *Solanum lycopersicum*	pDNA‐amiRNA	Leaf spraying	LDH clay nanosheets delivered pDNAs carrying amiRNAs into plant cells and silenced the virus‐produced transcripts.	[257]
CDs	Leaves of *N. benthamiana* and tomato	dsRNA	Leaf spraying	More than 80% reduction in GFP transcript and protein levels were measured.	[258]
CaP NPs	*Brassica juncea* L. cv. *Pusa Jaikisan*	pDNA	Co‐culture	The transformation efficiency was about 80% by using CaP‐based delivery platform compared to 54% by *Agrobacterium tumefaciens* and 8% by using naked DNA.	[259]

### 5.1 Carbon Nanotube‐Based Gene Delivery Platform

CNTs are long and thin cylindrical molecules that made of one or more layers of graphene sheets, which are divided into two types: SWCNTs and MWCNTs.^[^ ^226^ ^]^ Generally, the diameter of SWCNTs is less than 2 nm, and the diameter of MWCNTs is in the range of 5–100 nm.^[^ ^227^ ^]^ Their unique physicochemical properties improve their performance as ideal delivery tools of biomolecules. Their surface can also be functionalized to protect the biomolecules from degradation or denaturation, and the hollow interior can be filled with different biomolecules to be delivered. In addition, the unique nanotube‐like structures can be used as a highway for target molecules to be transported between cells. Some studies have demonstrated the ability of CNTs to deliver biomolecules in plant cells. For example, Liu et al. demonstrated that SWCNTs had the ability to penetrate the cell wall and cell membrane of intact tobacco BY2 cells, and further suggested that endocytosis might be the driving force for the uptake of SWCNTs by tobacco cells.^[^ ^209^ ^]^ Burlaka et al. found that SWCNTs and MWCNTs could successfully delivered DNA in plant cells after non‐covalent functionalization.^[^ ^228^ ^]^

**Figure 7:**
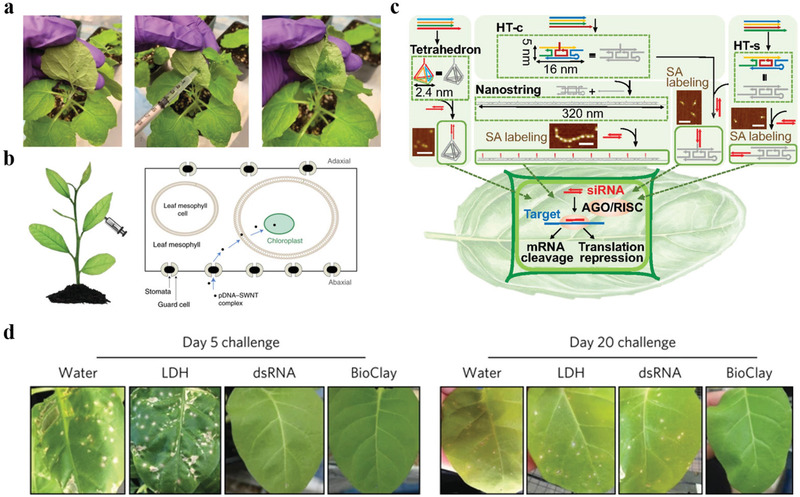
Nanomaterials‐based gene delivery platform. a) The infiltration of leaves with pDNA‐PEI‐SWCNTs. Reproduced with permission.^[^ ^230^ ^]^ Copyright 2019, Nature Publishing Group. b) The process of pDNA‐SWCNT complexes enter the mesophyll through stomata, traverse plant cell walls, plasma membranes, and finally enter the chloroplast bilayers. Reproduced with permission.^[^ ^39^ ^]^ Copyright 2019, Nature Publishing Group. c) Schematic diagram of the DNA nanostructure synthesis and the plant infiltration workflow. Reproduced with permission.^[^ ^213^ ^]^ Copyright 2019, National Academy of Sciences. d) Images showing the extent of necrotic lesions on *N. tabacum* cv. Xanthi leaves challenged with PMMoV after 5 days and 20 days post spray treatment. Reproduced with permission.^[^ ^252^ ^]^ Copyright 2017, Nature Publishing Group.

Ribose nucleic acid interference (RNAi) is a phenomenon induced by double‐stranded RNA (dsRNA) and sequence‐specific inhibition of gene expression at the level of messenger RNA, including transcriptional gene silencing or post‐transcriptional gene silencing.^[^ ^229^ ^]^ Based on RNAi, Demirer and colleagues developed a SWCNTs‐based nanocarrier for direct delivery of small interfering RNA (siRNA), and successfully silenced green fluorescent protein (GFP) gene expression in transgenic mGFP5 *Nicotiana benthamiana* (tobacco, Nb) plants.^[^ ^41^ ^]^ They injected Cy3 fluorescent dye‐tagged siRNA‐SWCNTs (Cy3‐siRNA‐SWCNTs) and ‐tagged free siRNA (Cy3‐siRNA) solutions into the intact plant leaves through a needleless syringe. They found that leaves infiltrated with Cy3‐siRNA‐SWCNTs exhibited high degrees of co‐localization between the intracellular GFP and Cy3 fluorescence channels than Cy3‐siRNA, demonstrating the effective internalization of siRNA‐SWCNTs. After infiltrating the leaves with siRNA‐SWCNTs, the expression of GFP in cells was significantly reduced. This study verified that SWCNTs could deliver siRNA and silence endogenous genes efficiently in intact plant cells, and they also could protect siRNA from nuclease degradation. The group also developed the high aspect ratio CNTs‐based platform for the delivery of plasmid DNA (pDNA) into mature plant cells in a species‐independent manner.^[^ ^38^, ^230^, ^231^ ^]^ This method enabled efficient and non‐toxicity delivery of pDNA without mechanical aid and transgene integration. The authors first covalently modified carboxylated CNTs (COOH‐CNTs) with a cationic polymer (poly‐ethylenimine, PEI), and further grafted negatively charged pDNA on the PEI‐CNTs through electrostatic grafting (pDNA‐PEI‐CNTs). The co‐localization of Cy3 fluorescence with GFP demonstrated the successful internalization of pDNA‐CNTs in plant (GFP mutant Nb plant) leaves after the delivery of Cy3‐tagged pDNA‐CNTs. And the formulation of pDNA‐PEI‐SWCNTs was more efficient than pDNA adsorbed on pristine MWCNTs via dialysis. In addition, strong GFP expression was observed in leaf lamina cells of all plant species tested (tobacco, wheat, arugula, and cotton leaves) after infiltrating leaves with GFP‐encoding pDNA (**Figure** **7**a). Thus, the authors demonstrated that CNTs could be useful tools for gene delivery.

In addition, Wong et al. established a lipid exchange envelope and penetration (LEEP) mathematical model, which was consistent with existing knowledge on chloroplast membrane structure, to study the impact of size and zeta potential of NPs on their ability to penetrate plant membranes.^[^ ^43^ ^]^ They found that the size and zeta potential had effects on the passive internalization of SWCNTs across the double lipid bilayer of chloroplasts. The model predicted a critical particle size, which NPs might unable to penetrate the membrane regardless of the zeta potentials when the size of NPs below this value. And there is also a minimum threshold zeta potential that NPs of a certain size must possess to penetrate the membranes. Based on this model, the physical and optical properties of nanomaterials can be adjusted to optimize the passive delivery of biomolecules, thereby crossing plant barriers that have hitherto been difficult to access. Based on this, Kwak et al. utilized the LEEP model to tune the properties of nanomaterials to deliver biomolecules to specific plant organelles.^[^ ^39^ ^]^ They designed several chitosan‐complexed SWCNTs (CS‐SWCNTs) through non‐covalent wrapping or covalent modification of the nanotube sidewall, where the functionalized SWCNTs could be used as cationic nanocarriers to load negatively charged pDNA through electrostatic interactions and release the pDNA selectively in the chloroplasts. According to the LEEP model, when the ratio of pDNA:SWCNTs was lower than 1:1 (w/w), the conjugates had a higher zeta potential than the threshold predicated by the model, thereby the SWCNTs could bind onto the anionic pDNA more tightly. When the ratio of pDNA:SWCNTs exceeded 1:1, physical aggregation of the SWCNTs was observed due to the weaker electrostatic repulsion between NPs with lower surface charge, resulting in a lower drive to penetrate the chloroplast envelope. They employed pDNA encoding YFP for transient expression in the chloroplasts, and observed the YFP expression in the chloroplasts of several plants, for example, mature tobacco, spinach, watercress, and *Eruca sativa* plants (Figure 7b). Lew et al. synthesized imidazolium (IM)‐functionalized SWCNTs (IM‐SWCNTs), a new type of nanocarriers that could traverse pollen barriers and deliver genes without the aid of external force.^[^ ^232^ ^]^ Compared with CS‐SWCNTs and PEI‐SWCNTs, which were used to deliver biomolecules to plant cells, the synthesized IM‐SWCNTs were more biocompatible toward pollen grains. According to the LEEP model, zeta potential is the main controlling factor for NPs to pass through the pollen plasma membrane, thereby the highly charged IM‐SWCNTs (larger than the threshold zeta potential) exhibit a better capacity to interact and pass through plant membranes. The author believes that this research provides insights for the design of novel nanocarriers, and whether this nanomaterials‐mediated gene delivery can be stably integrated into the genome of pollen cells will be the focus of future work.

From the aforementioned studies, such CNTs‐based gene delivery platform is more appropriate, time‐saving, and cost‐effective for delivering exogenous biomolecules. It is beneficial for plant biotechnology applications where gene expression without transgene integration is desired, thereby circumventing strict GMO regulations. And for the LEEP model, which is used for SWCNTs entry into the chloroplast, helping people understand the impact of size and zeta potential on their ability to penetrate plant membranes from a physical and mathematical perspective. However, further design is still needed to for using SWCNTs or other nanomaterials as possible nanocarriers into more plastids like the chloroplast. In addition, further optimization work to make the chloroplast stable transformation of crop species is also necessary. More importantly, before full promotion and application, multiple rounds of selection and breeding are carried out to ensure that SWNTs do not exist in the plant in order to meet the safe use and acceptability in agricultural applications. More importantly, multiple rounds of selection and breeding should be strictly carried out to ensure no trace of SWCNTs in plants before they can be fully promoted and applied, so as to satisfy the safe use and acceptability of such platform in agricultural applications.

### 5.2 Mesoporous Silica Nanoparticles‐Based Gene Delivery Platform

MSNs have been widely used as nanocarriers to deliver biomolecules in animal cells and tissues because of their biocompatible and biodegradable nature.^[^ ^233^ ^]^ Thanks to the presence of exposed silanol groups, they can be easily functionalized, which greatly expand their applications. In addition, MSNs also have rich texture properties, adjustable pore size (2–20 nm), and large specific surface area.^[^ ^234^ ^]^ Therefore, compared with traditional methods, the MSNs‐based gene expression system is considered to be a suitable alternative for transient transformation method and rapid assessment of gene function.^[^ ^235^ ^]^ For example, Chang et al. utilized MSNs functionalized with different organic groups to develop a MSNs‐mediated transient gene expression system, which could be used as nanocarriers to deliver biomolecules in *A. thaliana* without additional assistance.^[^ ^234^ ^]^ The authors labeled MSNs with fluorescein isothiocyanate (F‐MSNs), and further grafted the external surface of F‐MSNs with *N*‐trimethoxysilylpropyl‐*N*,*N*,*N*‐trimethylammonium chloride (TMAPS, positively charged) (TMAPS/F‐MSNs), which exhibited high binding ratio with pDNA (negatively charged). Results showed that the TMAPS/F‐MSNs‐based gene delivery platform could pass through the cell wall barriers of plants, and then reached the deep tissues (e.g., cortex and endodermis) and several organelles (e.g., plastids and nuclei) through a simple co‐culture method.

However, the aforementioned process is conducted in vitro conditions, which requires grand costs and special conditions like sterile culture. Therefore, under in vivo conditions, Hajiahmadi and coworkers utilized MSNs to deliver pPZP122:35S:GUS (pDNA) (pDNA‐MSNs) to the deep tissues of tomatoes through stomata and shoot.^[^ ^220^ ^]^ The MSNs was functionalized with aminopropyl triethoxysilane (positively charged), which exhibited high binding ratio and stability with pDNA. The pDNA‐MSNs transferred in plants mainly through three ways, including sprayed the solution on the abaxial surface of leaves, injected the solution with needleless syringe into the shoot and the abaxial surface of leaves. Results showed that by injecting the pDNA‐MSNs into the abaxial surface of leaves, the MSNs‐based transient expression system successfully transferred the exogenous genes into plants and further performed the transcription and translation steps in the target plant tissues. Although this method has unique advantages, the system still needs to be further optimized in future research to achieve stable plant transformation and further expand plant species.

### 5.3 Metal Nanoparticle‐Based Gene Delivery Platform

Metal NPs with the properties of optical absorption and scattering, giving them new application prospects in bioimaging and biomedical fields.^[^ ^32^, ^236^ ^]^ Among them, Ag NPs with the properties of adjustable size, easy synthesis and surface modification, and well‐defined surface chemistry have been widely used for the delivery of molecules in animals cells, such as DNA, drugs, and peptides.^[^ ^237^ ^]^ Although the use of metal NPs in plant sciences as nanofertilizers or nanopesticides for increasing crop yields have been widely reported, their application as nanocarriers in plants to deliver genes is still limited. Vijayakumar et al. realized the delivery of DNA into plants by using Au NPs that embedded in sharp carbonaceous carriers.^[^ ^221^ ^]^ The carbonaceous carriers with sharp edges could penetrate the rigid cell walls and nuclear membranes of plants, realizing the delivery of genes to the chromosomal. However, this gene delivery platform was assisted by a gene gun, which might cause tissue damage. Although there are few studies on the direct use of Au NPs for the delivery of biomolecules in plants without the help of external force at present, through further refinement of Au NPs, such as the development of monolayer‐protected Au NPs, polymers/Au nanocomposites, and other functionalized Au NPs, the Au NPs‐based biomolecule delivery can be realized in the future.

### 5.4 Magnetic Nanoparticle‐Based Gene Delivery Platform

MNPs possess magnetic properties, as well as, the general properties of metal NPs. The cores of common MNPs are made of functionalized Fe_2_O_3_ NPs, and the shells are made of inert gold layers, inert metal seeds layers, or silica layers.^[^ ^238^ ^]^ The small size of MNPs allows them to penetrate cells, and their superparamagnetic properties further enhance their ability to load biomolecules and transport biomolecules with the help of external magnetic field.^[^ ^238^, ^239^, ^240^ ^]^ In one study, Hao et al. synthesized Au‐coated MNPs (mGNPs) with uniform size and morphology, and further functionalized the mGNPs with PEG (mGNPs‐PEG), which added free amino functional groups.^[^ ^222^ ^]^ The mGNPs‐PEG were bound with fluorescein isothiocyanate (FITC) molecules and pDNA through the amide covalent bond, and further delivered the biomolecules into canola protoplasts with the help of an external magnetic field. They found that the green fluorescence of FITC was visible inside the cells, demonstrating the successfully delivery of mGNPs‐PEG‐FITC. For the gene delivery, results showed that blue color appeared in the canola protoplasts and walled canola cells, indicating the successful gene expression. The authors believed that the mGNPs could be developed as good nanocarriers to deliver biomolecules in plants in the further.

The concept of “pollen magnetofection” has been reported by Zhao et al. to deliver exogenous DNA into pollen grains for transient transformation, and the exogenous DNA can stably inherit to the next generation.^[^ ^241^ ^]^ The external magnetic field helped the delivery of MNPs‐DNA complexes into the pollen, and the magnetofected pollen was used for plant pollination, thereby producing transgenic seeds. However, the efficiency of magnetofection is directly controlled by the number and size of pollen apertures, which is difficult to realize the process when the pollen has a small and single aperture.^[^ ^32^ ^]^ Several researchers believed that the pollen magnetofection method described by Zhao et al. was only a potential gene transformation technique, and the transient transformation of pollen in several monocot plants (maize, sorghum, and lily) was not successful.^[^ ^242^ ^]^ Therefore, the stable transformation of plants through the pollen magnetofection still needs further research. Atleast, this pollen magnetofection method do not succeed in achieving transient pollen transformation in several monocot species.

### 5.5 DNA Nanostructure‐Based Gene Delivery Platform

A few decades ago, the idea of assembling simple DNA sequences into meaningful structures was proposed, which is also the origin of the modern DNA nanotechnology.^[^ ^243^ ^]^ The DNA nanotechnology provides new research directions for the design, synthesis, and utilization of novel nanomaterials. Based on the programmability of DNA Watson Crick base pairing, DNA nanostructures (DNs) are assembled into pre‐designed shapes through the sequence‐specific hybridization of template and staple DNA strands.^[^ ^244^ ^]^ Unlike separated DNA, the DNs with different sizes and shapes can be easily taken up by cells through caveolin‐ or clathrin‐mediated endocytosis, and have been widely applied in drug, DNA, RNA, and protein delivery.^[^ ^245^, ^246^, ^247^, ^248^, ^249^ ^]^ Although their application in plant systems has not been widely studied, considering that DNA is the basic element of plants, DNs are biocompatible, nontoxic, and easy to be metabolized. They may have great potential in plant genetic engineering.

In one study, Zhang et al. synthesized novel DNs and first tracked the DNs in the plant cell cytoplasm of some species including Nb, *N. tabacum*, *E. sativa*, and watercress (Figure 7c).^[^ ^40^, ^213^ ^]^ The DNs were further utilized to load siRNA targeting a GFP gene and infiltrated into the leaf abaxial side (transgenic mGFP5 Nb). Without the help of external aid, the gene silencing efficiency in leaves was in line with the internalization trends of DNs in plant cell cytosol. The leaves infiltrated with siRNA‐DNs exhibited the large decrease in GFP fluorescence intensity. The shape, size, compactness, and stiffness of DNs and the siRNA attachment locus might affect the silencing mechanism of plant endogenous gene. Therefore, this study first demonstrates the ability of plant cells to internalize a series of DNs with exogenous biomolecules, which only has been proven to be valuable in animal systems. Such DNs‐based gene delivery platform that enables siRNA delivery and gene silencing without damaging plant tissue, and further set guidelines for the design and application of various DNs that can be effectively taken up into plant cells.

### 5.6 Other Nanomaterial‐Based Gene Delivery Platform

Besides the aforementioned nanomaterials, some studies also have demonstrated the possibility of other nanomaterials, such as, layered double hydroxide (LDH), CDs, etc., as nanocarriers to deliver biomolecules to plants. LDH clay nanosheets are a family of inorganic layered materials with the properties of biocompatibility and biodegradability, which have been utilized for the delivery of biomolecules in cells.^[^ ^250^, ^251^ ^]^ Mitter and coworkers synthesized novel biodegradable LDH clay nanosheets as a nanocarrier for the delivery of dsRNA in plants.^[^ ^252^ ^]^ For plants, topical application of pathogen‐specific dsRNA to control viruses through the RNAi‐mediated systemic protection to replace genetically modified plants is a meaningful study.^[^ ^253^, ^254^, ^255^, ^256^ ^]^ But the naked dsRNA is unstable after spraying on plants. In view of this, the authors loaded dsRNA on cationic LDH through the electrostatic interactions and found that the dsRNA did not wash off after applying the dsRNA‐LDH (bioClay) to the surface of *Arabidopsis* leaves. They revealed that the dsRNA in bioclay was protected from nuclease activity and was successfully delivered in plant cells. And since the atmospheric CO_2_ and humidity will form carbonic acid on the leaf surface, the LDH nanosheets could be completely degraded. Results showed that the bioClay could provide a longer protection window to plant leaves than naked dsRNA (Figure 7d). Liu and coworkers prepared LDH clay nanosheets loaded with pDNA expressing artificial microRNA (amiRNA) for efficiently preventing Tomato yellow leaf curl virus (TYLCV) infection in tomato plants, which was realized via the amiRNA‐mediated silencing technology.^[^ ^257^ ^]^ Results showed that the LDH clay nanosheets successfully realized the delivery of pDNA carrying amiRNA into plant cells and silenced the virus‐produced transcripts in Nb and *Solanum lycopersicum* plants. Compared with the control groups, the disease severity and the concentration of TYLCV of the plants treated with pDNA‐LDH were significantly reduced. The authors demonstrated that the pDNA‐LDH clay nanosheets might be a useful tool to improve plant resistance to TYLCV.

Since it has been proven that RNAi can be used to silence specific genes to enhance the resistance of plants to pathogens, viruses, and insects, some applications have been implemented in agriculture. With a deepening understanding of the RNAi pathway, the most commonly used methods were the use of dsRNA and amiRNA mentioned above. And the use of topically applied RNAi effectors is beneficial to plant functional genomics. Besides the aforementioned methods that utilized LDH clay nanosheets to deliver RNAi effectors, Schwartz and coworkers first realized the delivery of dsRNA in plant cells (*Nb* and tomato leaves) by using CDs.^[^ ^258^ ^]^ After the topical spray application of the dsRNA‐CDs, the GFP transgenes in both species achieved strong silencing, and the GFP transcript and protein levels were reduced by more than 80%. The authors revealed that the amine functionalized CDs (positively charged) could bind to the negatively charged dsRNA, protecting the dsRNA from degradation by nucleases and enhancing the cellular uptake of dsRNA. In addition, the small size of the synthesized CDs (≈3.9 nm) was also a prerequisite for dsRNA to effectively pass through plant cell walls.

For other nanomaterials‐based gene delivery platform, Naqvi et al. synthesized calcium phosphate (CaP) NPs for the delivery of pDNA (pCambia 1301) into the plant nuclei (*Brassica juncea* L. cv. *Pusa Jaikisan*).^[^ ^259^ ^]^ Results showed that the transformation efficiency was about 80% by using CaP NPs‐based delivery platform compared to 54% by *Agrobacterium tumefaciens* and 8% by using naked DNA. Sarker and coworkers prepared functional nanomaterials (Arabinoxylan (AX)) from wheat bran derived polysaccharide and utilized the AX to deliver biomolecules.^[^ ^260^ ^]^ After chemical modification, positively and negatively charged domains were introduced to AX backbone, inducing the local electrostatic/hydrophobic interactions and promoting the formation of NP structures (AX NPs). Results showed that the positively charged AX NPs could complex with negatively charged CRISPR‐Cas9 DNA. The authors revealed that the biodegradable AX NPs could be used as useful nanocarriers for the gene delivery in plants.

## 6 Nanogenerators Based on Plant

**Figure 8:**
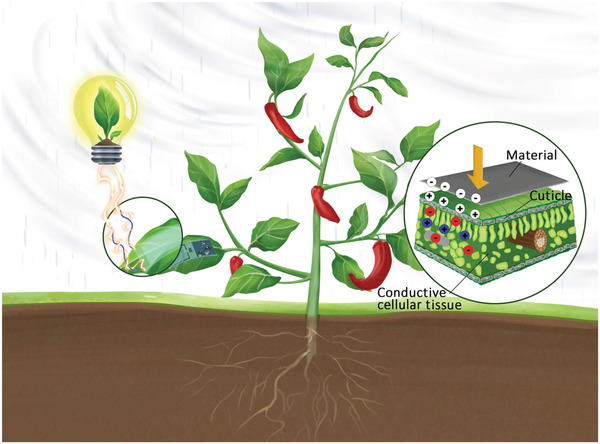
Nanogenerators based on plant.

According to research, the energy demand would grow by 32% to reach 17934 million tons of oil equivalent in 2040.^[^ ^261^ ^]^ This will require a significant increase in oil production and further increase in carbon dioxide emissions, leading to a great threat to the environment. In this case, in addition to reducing the waste of unsustainable resources, the development of green and sustainable energy is very necessary. Plants are widespread in nature, and when we talk about electricity produced by plants, we often think of the power from photosynthesis and bioelectricity. But in fact, plants have greater application prospects in the field of energy development, which still requires a lot of researches. And the energy conversion by plants is intrinsically sustainable, which is closely related to the sustainable green energy that people urgently need in the future.^[^ ^262^ ^]^ As shown in **Figure** **8**, environmental mechanical energy widely exists in our daily life, such as wind, rain, waves, vibration, etc., although most of this energy is neglected and wasted. However, when plants are blown by the wind, like flowers flap in the breeze, they are actually utilizing the kinetic energy of the wind. Although the amount of energy collected by a plant is rather small, there are plenty of plants in the environment. Therefore, the development of these ubiquitous environmental micro‐energy that can be harvested by plants is of great research value.

TENG is based on the principles of triboelectrification and electrostatic induction, which can effectively collect mechanical energy in the environment to provide power for electronic devices.^[^ ^263^ ^]^ The basic structure of TENG consists of conducting electrodes and two triboelectric layers that have different electronegativities. Compared with traditional mechanical energy harvesting devices based on piezoelectric or electromagnetic effects,^[^ ^264^, ^265^, ^266^ ^]^ TENG has the unique advantages of abundant triboelectric layer materials selection, high energy‐conversion efficiency, lightweight, flexibility, low cost, and simple structure. It should be noted that most of the TENGs that have been reported are constructed based on non‐biocompatible synthetic polymers, which limits their broad applications. Therefore, TENGs made of natural bio‐derived materials with advantages like low cost, renewability, and environmental friendliness, have received increasingly attention.

**Figure 9:**
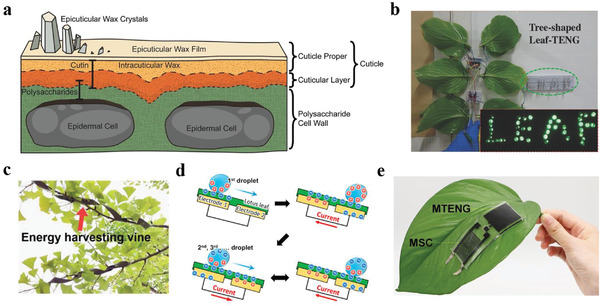
Using plants to capture mechanical energy from the environment. a) Schematic diagram of the major structural features of the cuticle and underlying epidermal cell layer. Reproduced with permission.^[^ ^268^ ^]^ Copyright 2013, American Society of Plant Biologists. b) Tree‐shaped energy harvester assembled with natural Leaf‐TENG. Reproduced with permission.^[^ ^47^ ^]^ Copyright 2018, Wiley‐VCH. c) The photo of energy‐harvesting vines. Reproduced with permission.^[^ ^274^ ^]^ Copyright 2018, Wiley‐VCH. d) The discrete liquid–solid contact electrification on the natural lotus leaf surface. Reproduced with permission.^[^ ^48^ ^]^ Copyright 2017, Elsevier Ltd. e) Photo of an integrated self‐charging device attached to the crop leaf. Reproduced with permission.^[^ ^49^ ^]^ Copyright 2020, Elsevier Ltd.

Plants are widely distributed in nature, and their leaves are the most common biomaterials in our life. The leaf surfaces possess an extracellular hydrophobic layer, that is, the cuticle, which is composed of a covalently linked macromolecular scaffold of cutin and a variety of organic solvent‐soluble lipids.^[^ ^47^, ^267^, ^268^ ^]^ And the mixture of lipids are collectively called waxes. The microscopic structure of the cuticle is often divided into two parts: A cutin‐rich area rich in polysaccharides (the “cuticular layer”) and an covering layer rich in waxes but low in polysaccharide content (the “cuticle proper”) (**Figure** **9**a).^[^ ^268^ ^]^ During the formation of the cuticle, the wax either accumulates on the surface of cuticle as epicuticular wax crystals or films, or deposits within the cutin matrix.^[^ ^269^ ^]^ The wax is composed of various organic compounds, which endow wax with unique physical and chemical properties.^[^ ^270^ ^]^ More importantly, the epicuticular waxes with abundant biological microstructures are conducive to increasing the contact area between materials, thereby promoting the generation of electric charges in the process of triboelectrification.^[^ ^271^, ^272^, ^273^ ^]^ The internal leaf tissue (including the mesophyll, xylem, phloem, and apoplast) between the upper and lower cuticles, which has abundant water and electrolyte inside and can transport charges throughout the plant by ionic conduction.^[^ ^262^ ^]^

Based on the above introduction to the composition of plant leaves and their unique properties, it is possible to use leaves as a substitute for synthetic polymers to assemble TENG. Yang and colleagues first utilized Hosta leaf as the electrification layer and electrode to assemble TENG (Leaf‐TENG), and the poly(methyl methacrylate) (PMMA) was chosen as another contact layer.^[^ ^47^ ^]^ The authors found that the abundant microstructure of the leaf cuticle greatly increased the contact area between the leaf and PMMA in the process of triboelectrification, and the internal leaf tissue that rich in water and electrolyte can be used as conductive liquid. The maximum output power was about 45 mW m^−2^, which could drive the LEDs and electronic temperature sensors. In addition, a tree‐shaped energy harvester was constructed by connecting the Leaf‐TENG in parallel to harvest a large area of environmental mechanical energy (Figure 9b). Meder and colleagues further confirmed the possibility of using the entire plants to harvest natural mechanical stimuli (such as wind) and convert the stimuli into electricity by the triboelectric mechanism, which could also call the entire plants as “living triboelectric energy harvesters.”^[^ ^262^ ^]^ They first revealed how charges generated, accumulated, and compensated on living plant leaves, and verified that the cuticle of plant leaf could be used as a dielectric layer on top/bottom of the ion‐conductive cellular tissue. In another study, Kim et al. first revealed that the natural lipids on natural substances (e.g., leaves, hair, skin, and cells) might lose electrons and generate positive static electricity during triboelectrification, which shows the potential of fabricating lipid‐based TENGs.^[^ ^274^ ^]^ Since the lipids of living leaves can regenerate within hours after they have been exfoliated.^[^ ^275^ ^]^ They exfoliated the regenerative lipids from leaves with adhesive tape and contacted/separated with a silicone rubber. The output current was about 50–65 µA and voltage was about 200–220 V. They further proposed a new concept of “energy harvesting vines” by wrapping the artificial vines (a multilayer structure of silicone rubber/conductive fabric/silicone rubber) on tree branches, and electricity was produced under the contact/separation process between the vines and leaves under wind energy (Figure 9c). Feng et al. used abandoned dry leaves as the frictional materials to fabricate the TENG.^[^ ^276^ ^]^ Results showed that when the dry leaf‐based friction layers were rubbed with poly(vinylidene fluoride), the output current and voltage of the dry leaf‐based TENG could reach 25 µA and 560 V, respectively. Such new type of TENG based on dry leaf proposed exhibit unique application prospects of waste dry leaf in daily life, which provide a new direction for future researches.

The above studies have confirmed the potential of using plant leaves to construct TENG, which can well harvest the wind energy in the environment. In addition to the wind energy, plants can also harvest raindrop energy through the contact and separation process between raindrops and leaves. Since many surfaces in nature possess epicuticular wax that exhibits hydrophobicity, such as lotus leaf. When water droplet falls on the lotus leaf, it immediately roofs off from the surface. The whole process can be regarded as the sequential process of contact and separation between water droplets and solid surfaces, which will generate net charges (Figure 9d).^[^ ^48^ ^]^ Therefore, Choi and colleagues first utilized the discrete liquid–solid contact electrification and fabricated the TENG based on lotus leaf (LL‐TENG), proving that the lotus leaf could harvest neglected but sustainable energies in nature.^[^ ^48^ ^]^ Based on this, our group utilized LIG technology to fabricate an integrated self‐charging device with lotus leaf‐like bionic surface.^[^ ^49^ ^]^ This device was consisted of a multi‐operating mode TENG (MTENG) and a micro‐supercapacitor (MSC), and the design of bionic structure endowed the device with waterproof and self‐cleaning capabilities. The device could be attached to plant leaf surface for harvesting the triboelectric energy generated by the falling raindrops, and converted it into electric energy for storage and use. As shown in Figure 9e, the MSC could be charged by driving the MTENG in water‐dropping modes, which mimicked the process of raindrops falling. Besides lotus leaf, some petals also have hydrophobic properties. Chen et al. demonstrated that the rich micro‐ and nanostructures on the surface of rose petal provide them with sufficient roughness and hydrophobicity, and constructed the TENG by using rose petal and PMMA.^[^ ^267^ ^]^ They used deionized water to imitate rainwater, and found that the sequential process of contact/separation of water droplets from the petal surfaces would generate electrical charge, where the peak of output current was 7.84 nA. All in all, the energy generated by raindrops striking flowers or leaves in rainy days can be electrified, which represents a new way to harvest energies previously unknown in nature.

From the above studies, it is not difficult to find that the use of TENG technology has greatly developed the potential of plants to develop new energy. These micro‐energies, which are easily ignored, are well collected and utilized. Moreover, compared with large‐scale photovoltaic power generation or wind power generation devices, which are expensive, subject to weather restrictions, some of the raw materials used are not environmentally friendly, and the locations are fixed. The use of plants to construct TENGs is small, environmentally friendly, portable, and the energy generated is continuous. Therefore, they can serve as a complement to the existing energy development devices to jointly develop sustainable new energy.

## 7 Nanotoxicology on Plant Health

**Figure 10:**
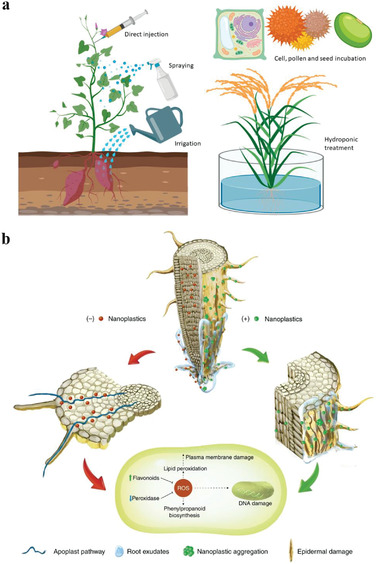
The possible adverse effects of nanomaterials in plants. a) Different ways that plants are exposed to nanomaterials. Reproduced with permission.^[^ ^50^ ^]^ Copyright 2020, Multidisciplinary Digital Publishing Institute. b) Schematic illustration of the uptake of nanoplastics in an *Arabidopsis* root and the plant defense mechanisms that are induced. Reproduced with permission.^[^ ^295^ ^]^ Copyright 2020, Nature Publishing Group.

Although nanomaterials have been widely used in the modern agriculture such as various applications that mentioned above, we still do not fully understand the possible adverse effects of nanomaterials in the environment, especially in plants and organisms. Therefore, the study of nanotoxicology is of great importance for understanding the interaction mechanisms between nanomaterials and living organism. Several researchers have explored whether nanomaterials have adverse effects on the normal growth of animals, microorganisms, cells, etc.^[^ ^277^, ^278^, ^279^ ^]^ Especially for plants, studies on the uptake, transformation, and toxicity of nanomaterials on plants are important. As shown in **Figure** **10**a, there are different exposure pathways of nanomaterials, such as injecting nanomaterials into plant tissue through leaves, sprying nanofertilizers, and nanopesticides on leaves or soils, contaminating the soil with nanomaterials, etc.^[^ ^50^, ^280^, ^281^ ^]^ The classic toxicology usually holds the view that “the dose makes toxicity,” that is, the concentration of a material determines whether it is dangerous.^[^ ^50^ ^]^ However, in fact, besides the concentration of nanomaterials, the size, surface area, surface charge, morphology, modification, surface chemistry, and aggregation of nanomaterials are all related to their toxicity level.^[^ ^282^ ^]^

Since the morphological and physicochemical properties of nanomaterials may have great effects on the interaction with plants, many toxicity tests have been performed. From aforementioned studies, CNTs have been widely applied in plants. There are many reports on the toxicological and physiological effects of CNTs on plants, and the main mechanism of toxicity for CNTs is that they may cause cellular oxidative stress and metabolic disorders in plants.^[^ ^283^, ^284^ ^]^ For example, Giraldo et al. demonstrated that SWCNTs could passively enter the chloroplasts extracted from spinach leaves and further irreversibly localized within the lipid envelope, which had effects on the photosynthetic activity, and the content of chlorophyll and ROS.^[^ ^98^ ^]^ Hatami et al. demonstrated that CNTs with different rate‐dependent concentrations could cause metabolic disruption of vascular plants.^[^ ^285^ ^]^ And Jordan et al. studied the physiological responses of plants to CNTs by growing tomatoes on soil contaminated by CNTs.^[^ ^286^ ^]^ They found that only MWCNTs had effects on the time of first fruit, and SWCNTs had greater effects on the physiology of tomatoes, where the content of salicylic acid greatly increased. And although the CNTs affected the growth and fruiting time, they have no effects on the total fruit yield of tomatoes. These findings do confirm that CNTs have more or less impacts on the physiological processes of plants. However, no general concept of the interaction between CNTs and plants has been given. And there are no long‐term experiments, because the enrichment of these nanomaterials in plants or soil requires several years of follow‐up studies to verify their effects on environment and living organism.

Plastics are an integral part of everyday life. The contamination of the environment with nanoplastics (<100 nm) and microplastics (0.1–5 mm) have emerged as a global challenge because they may pose risks to biota and public health.^[^ ^287^, ^288^ ^]^ Although the potential risks of microplastics in aquatic ecosystems have been well studied, little is known about their impacts on the terrestrial environment.^[^ ^288^, ^289^, ^290^ ^]^ Weithma et al. studied the potential of organic fertilizers as possible carriers for microplastic particles to enter the environment through the fermentation and composting of biological wastes.^[^ ^287^ ^]^ They first proved that organic fertilizers may be a source of microplastics particles in the environment, which should not be ignored. Since land‐based sources can be considered as a long‐term storage place for micro‐ and nanoplastics, plants may easily take up them, and transfer them to shoots and eventually accumulate in roots.^[^ ^291^, ^292^, ^293^, ^294^ ^]^ Sun and coworkers prepared two kinds of functionalized polystyrene (PS) nanoplastics (PS‐SO_3_H (55 ± 7 nm) and PS‐NH_2_ (71 ± 6 nm)), which were negatively (−46.8 ± 1.8 mV) and positively (+28.1 ± 2.0 mV) charged, respectively.^[^ ^295^ ^]^ They planted *A. thaliana* in soils that mixed with either PS‐SO_3_H or PS‐NH_2_. Results showed that the growth and seedling development of *A. thaliana* exposed to different concentrations of PS‐SO_3_H and PS‐NH_2_ were inhibited compared with controls, where the effects of positively charged nanoplastics were greater than negatively charged nanoplastics. And both nanoplastics treatments reduced plant disease resistance, where the disease resistance genes of plants were downregulated. They further utilized fluorescently labelled nanoplastics to study how plants took up them, and found that positively charged nanoplastics accumulated in the root tips, while negatively charged nanoplastics accumulated in the apoplast and xylem (Figure 10b). Moreover, the uptake and internalization of positively charged nanoplastics were lower than that of negatively charged nanoplastics.

The findings help us better understand the effects of nanoplastics on terrestrial ecosystems, and have great reference significance for studying the uptake and transformation of other nanomaterials in plants and their effects on the physiological processes. From the above several chapters, we find that, compared with animal cells, only a small number of NPs can penetrate plant cells. Such a limited intake process is usually due to that plants not only have a nanoscale thickness phospholipid bilayer cell membrane, but also a rigid cellulose cell wall and a co‐extensive pectin network that can reach several microns. These nanomaterials that can penetrate plant cells require extensive and long‐term studies to prove their uptake, transformation, and ultimate fate in plants.

## 8 Conclusions and Perspectives

Nanotechnology, which is the production and manipulation of matter at length scales with at least one dimension of nanoscale, has been widely developed and applied in agriculture to promote plant growth and yield. These include the dynamic and real‐time monitoring of plant growth process at the micro‐nano scale, nutrient and pest management, promotion of plant transformation, environmental protection, and development of plants’ potential for harvesting environmental micro‐energy. Admittedly, these nanotechnologies do have many unexpected benefits with the in‐depth understanding of “plant nanoscience.” However, there are still many challenges in the future, including unified international standard, fragmented policy, nanosafety issues, health of growers, social perception and acceptance, “real‐life” application, etc.

As mentioned above, the number of publications and patents of nanotechnology in the agricultural field is increasing. The large‐scale scientific research and the application of commercial products are not yet available due to a number of reasons, including inconsistent national legislative frameworks, limited regulation, a lack of public licensing initiatives, etc. In addition, the physicochemical and biological properties of nanomaterials are different from single atoms, molecules or bulk materials in many aspects, which makes it difficult to predict the direct/indirect and cumulative effects of nanomaterials. More importantly, the migration of nanomaterials in the environment, organisms, and humans is still inconclusive. These problems greatly hinder the formulation of internationally unified policies and regulations. Thus, cooperation between international and national organizations is indispensable, and a great deal of research and field work needs to be carried out immediately. The successful application of nanotechnologies in agriculture will only be possible by working together to overcome the challenges posed by ununified policies and regulations.

Since agriculture is a complex ecosystem, the use of nanomaterials and nanotechnology may pose a large exposure risk, and there is still a disconnect in the current research on the risks of nanomaterials to human and environmental health. For CNTs, graphene, metal NPs, zero‐valent iron and other nanomaterials mentioned above, which have been used to control the release of agrichemicals, targeted delivery of biological molecules, environmental remediation, etc. The study on the specific interaction mechanism between them and the target, especially the complex plant system is still not deep enough. These applications will add a considerable degree of complexity to the multifaceted risk and exposure that already exist. Therefore, when using any nanomaterials and nanotechnology, nanosafety issues need to be taken seriously. In the process of experimental design, the selection of materials should be careful, and natural materials with very little threat to the health of growers and consumers should be considered. Then, the influence of special nanomaterial properties (e.g., shape, size, and charge) on the interaction with different plants needs to be carefully investigated. At last, it is necessary to understand the transformation and fate of nanomaterials in the environment to assess potential risks.

Although these nanotechnologies have exhibited unique advantages different from those traditionally used in agriculture, they are still primarily at the research stage. The results mentioned above have been repeatedly verified in the laboratory, but there are very few cases showing that they have moved from the laboratory to the field scale, and even fewer examples of commercial applications. More importantly, most of the research on plant systems is based on previous work, such as the monitoring of animals. These studies may only show the great potential and influence of their application to plant systems. For example, metal NPs, nano‐chitosan, nano‐zeolite, etc., have been proven to improve uptake efficiency and target active ingredients to specific plant cell compartments and organelles. They still need to be carefully designed according to different soil conditions and plants, and formulate a unified concentration standard. And, scale up the delivery of nanomaterials under various field trials is also important. The transformation from laboratory to field needs to be realized as soon as possible. Moreover, long‐term experimental observations are needed to draw general rules to prove that these nanomaterials are indeed beneficial to plant growth and do not pose a threat to the health of animals and humans. It takes time from basic research to the stage that can be used for commercial applications, where the security, price, acceptability, etc. need to be considered.

The sustainability of nanotechnologies in agriculture also depends on social perception and acceptance. Many consumers still reject genetically modified foods and cannot accept nanotechnology in food. Therefore, involving consumers in the actual production process may help them have a better understanding of how these nanomaterials are produced and used. And the results of field trials must be conveyed to people in an unbiased and true manner. At last, it is necessary to collect more data on product safety and risk, as well as data on consumer acceptance of nanomaterials in food production. Through professional explanations, let the public understand nanosafety issues in detail, and finally accept food produced by using nanotechnology.

Only by truly addressing these challenges can nanotechnology promote to agricultural production. In the future, it is necessary to further strengthen the interaction between plants and nanomaterials on the basis of existing studies. Strano's research group first proposed the concept of plant nanobionics in 2014, that is, the interface between plant organelles and non‐biological nanostructures has the ability to give organelles new and enhanced functions.^[^ ^98^ ^]^ However, these functions are achieved by injecting SWCNTs into plants through the leaves. Whether the symbiosis between nanomaterials and plants can be realized in the future, that is, nanomaterials exist at the beginning of plant life and disappear with the end of plant life. This goal may be a redefinition of the concept of plant nanobionics, which provides a new direction for “plant nanoscience” research. Thus, how plant cells control gene expression by integrating external and internal factors, how plants feel the environmental stimuli for their survival, and how environmental stimuli regulate and determine plant physiology, growth, and morphogenesis may be intuitively reflected through these nanomaterials. These nanomaterials endow plants with non‐native functions and their dynamic changes can be observed by optical or electrical techniques, which indirectly reflect the signal transduction process of plant cells. In this way, timely measures can be taken to promote plant growth and increase production. But more importantly, these nanomaterials are harmless to plants and pose no threat to the health of animals and humans.

## Conflict of Interest

The authors declare no conflict of interest.

## Author Contributions

Q.Z. led the writing of this manuscript. J.P. and Y.Y. contributed to critical reading of the manuscript and the reviewing of appropriate references.
